# Supplementary material for: DeepNull models non-linear covariate effects to improve phenotypic prediction and association power
Source: Nat Commun. 2022 Jan 11;13:241. doi: 10.1038/s41467-021-27930-0 (PMC8752755; doi:10.1038/s41467-021-27930-0)
Supplement: Supplementary file 1 — Supplementary Information [file 41467_2021_27930_MOESM1_ESM.pdf]

DeepNull models non-linear covariate effects to improve phenotypic prediction and association power

Zachary R. McCaw[1], Thomas Colthurst[2], Taedong Yun[2], Nick Furlotte[1], Andrew Carroll[1], Babak Alipanahi[1], Cory Y. McLean[2,*], Farhad Hormozdiari[2,*]

1 Google Health, Palo Alto, CA, USA

2 Google Health, Cambridge, MA, USA

These authors contributed equally: Zachary R. McCaw, Thomas Colthurst

These authors jointly supervised this work: Cory Y. McLean, Farhad Hormozdiari

* Corresponding author: cym@google.com, fhormoz@google.com

Supplementary Notes

Supplementary Notes

Correct model specification increases association testing power.

To demonstrate theoretically that more accurately approximating the phenotype-covariate relationship should improve power, suppose that the generative model is:

$$Y_i = \beta_G G_i + h(X_i) + \epsilon_i.$$

Here Y_i is the phenotype of subject i, G_i is the genotype, X_i is a set of non-genetic covariates, $h(\cdot)$ is an unknown function, and ϵ_i is a residual with mean 0 and variance σ^2. The power to reject the $H_0 : \beta_G = 0$ is related to the non-centrality parameter (NCP) of the χ^2 test of association. The NCP is directly proportion to the sample size, and inversely proportion to the residual variance σ^2. Intuitively, as σ^2 increases, the power to reject the $H_0 : \beta_G = 0$ decreases.

Now, because $h(\cdot)$ is unknown, in practice we fit the working model:

$$Y_i = \beta_G G_i + h^*(X_i) + \epsilon_i,$$

where $h^*(X_i)$ is typically some simple function, such as $h^*(X_i) = \beta_{X1} X_i + \beta_{X2} X_i^2$. We can write the working model as:

$$Y_i = \beta_G G_i + h(X_i) + \epsilon_i^*,$$

where $\epsilon_i^* = h^*(X_i) - h(X_i) + \epsilon_i$. The residual variance of the working model is $\mathbb{V}(\epsilon_i^*) = \mathbb{V}\{h^*(X_i) - h(X_i)\} + \sigma^2$. Observe that the residual variance is increased by a term $\mathbb{V}\{h^*(X_i) - h(X_i)\}$ that depends on the discrepancy between the true $h(\cdot)$ and the working $h^*(\cdot)$. If $h^*(\cdot)$ is in fact $h(\cdot)$, then the working model is correctly specified, $\mathbb{V}\{h^*(X_i) - h(X_i)\} = 0$, and the test of $H_0 : \beta_G = 0$ has optimal power. However, when $h^*(\cdot) \neq h(\cdot)$, the residual variance of the working model increases, and the power to reject $H_0 : \beta_G = 0$ decreases. Thus, the more closely $h^*(\cdot)$ approximates $h(\cdot)$, the better the power. DeepNull uses a flexible DNN to learn $h^*(\cdot)$. By more closely approximating $h(\cdot)$

than the simple quadratics often used in practice, DeepNull is expected to reduce residual variation and thereby improve power.

DeepNull is efficient and robust across sample sizes.

To assess the computational overhead that DeepNull adds to association testing, we monitored the average time required for the DeepNull DNN to perform one fold of training in a five-fold cross-validation. All experiments were performed on commodity CPU hardware, and in each case DeepNull took 35 min to train. In addition, we observed little difference in DeepNull run times for different $f(\cdot)$ functions.

We varied the sample size n to assess its effect on DeepNull results. We simulated phenotypes for 20K, 50K, 100K, and 200K individuals under the single genetic architecture with $\sigma_g^2 = 0.4$ and $\sigma_x^2 = 0.4$, and considered the two non-linear functions $\exp(x)$ and $\text{sigmoid}(x)$ for $f(\cdot)$ in Equation (9). We observed that both the power and the expected χ^2 statistics for causal variants increase with the sample size (Supplementary Figure 2) while the type I error is controlled in all cases.

DeepNull results are not affected by initialization.

It is known that the initial random values assigned to weights at the beginning of training can influence deep learning methods, drastically in some cases. In this section, we investigate whether this phenomenon affects our DeepNull results.

We considered the genetic architecture where we set $\sigma_g^2 = 0.4$ and $\sigma_x^2 = 0.4$ and then simulated phenotypes for 10,000 individuals using UKB genotypes and covariates. We considered the two non-linear functions of $\exp(x)$ and $\text{sigmoid}(x)$ for $f(\cdot)$ in Equation (9). We ran DeepNull prediction using 100 different random seeds for each non-linear function. Then, for each pair of random seeds, we computed the Pearson correlation of the DeepNull predictions for all individuals. As depicted in (Supplementary Figure 3), those correlations ranged between 0.992 and 1.0. Thus, we conclude that DeepNull results are not significantly affected by the random initial seed.

Additional covariate adjustment simulations

Independent Covariate: If genotype $\bar{g}_i$ and the covariate x_i are independent, then adjusting for the covariate x_i is not necessary for unbiased estimation of the genetic effect β. However, adjustment

for x_i does improve efficiency, whether by directly including x_i in the association model, or by indirectly including x_i through $h(x_i)$. This is demonstrated in Supplementary Figure 26. The genotype only model $y_i = \bar{g}_i\beta + \epsilon_i$ is unbiased for estimating β, but has higher variance than the remaining models. In the absence of association between $\bar{g}_i$ and x_i, the unadjusted model (2), the linearly adjusted model (3), and the generative model are all equivalent.

Cubic Phenotype: For the quadratic phenotype considered in the main text, the estimate of β from the linearly adjusted model

$$y_i = \bar{g}_i\beta + x_i\gamma_1 + h(x_i)\gamma_h + \epsilon_i$$

is in fact numerically identical to that from the generative model

$$y_i = \bar{g}_i\beta + x_i\gamma_1 + x_i^2\gamma_2 + \epsilon_i.$$

To evaluate how the linear adjustment approach performs as the complexity of the relationship between y_i and x_i increases, we repeated the simulation with phenotypes generated from a cubic model

$$y_i = \bar{g}_i\beta + x_i\gamma_1 + x_i^2\gamma_2 + x_i^3\gamma_3 + \epsilon_i.$$

The simulation parameters were $\gamma_1 = 2$, $\gamma_2 = -1$, $\gamma_3 = 1/2$, with correlation 0.5 between $\bar{g}_i$ and x_i. As before, the prediction of y_i from x_i was obtained from the oracle model

$$y_i = x_i\gamma_1 + x_i^2\gamma_2 + x_i^3\gamma_3 + \epsilon_i,$$

and the two candidate models for estimating β were the unadjusted model (2) and the linearly adjusted model (3).

Results for the cubic simulation (Supplementary Figure 27) were nearly identical to those from the quadratic simulation (Figure 4). The estimate from the unadjusted model remained biased. While the estimate from the linearly adjusted model was no longer identical to that from the generative model, the difference was negligible.

Supplementary Figures

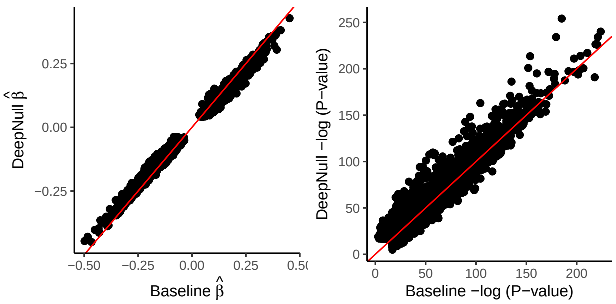

Supplementary Fig 1: **DeepNull reduces phenotypic residual variation.** a) Estimated effect size where the X-axis is the Baseline and Y-axis is the DeepNull estimated effect size. b) -log of p-value where the X-axis is the Baseline and the Y-axis is the DeepNull significant p-value. Both Baseline and DeepNull p-values are computed using two-sided tests. Each black dot represents one variant. The red diagonal line indicates the $y = x$. In both panels, we considered all variants that are genome-wide significant by Baseline or DeepNull. We use the simulated setting where $\sigma_g^2 = 0.4$, $\sigma_x^2 = 0.4$, and $f(\cdot) = \exp(x)$.

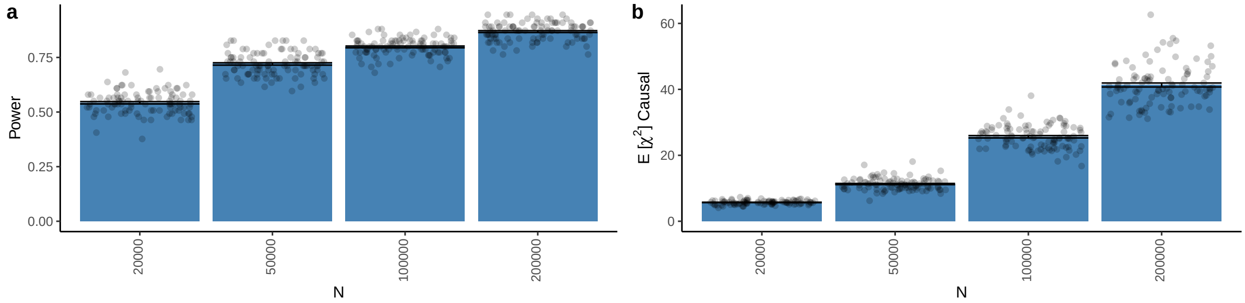

Supplementary Fig 2: **DeepNull results improve as sample size increases.** a) Y-axis is the statistical power of association. b) Y-axis is the expected χ^2 on the causal chromosome (`chr22`). Error bars are the standard error of the mean for each estimate and each bar plot summarises results from $n = 100$ independent simulation replicates. In both panels, the X-axis is the sample size in the simulated study. The numerical results are shown in Supplementary Table 7.

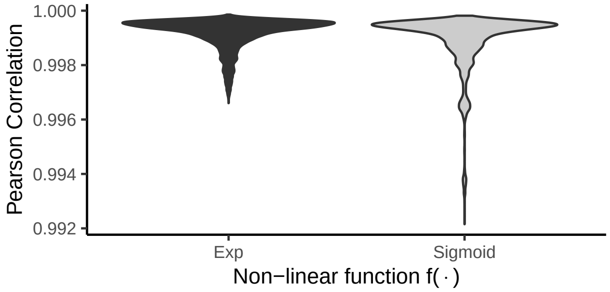

Supplementary Fig 3: **DeepNull results are not affected by random seed initialization.** Violin plot showing the distribution of Pearson correlations between the DeepNull DNN predictions for pairs of random seed initializers. The narrow range of correlation values shows that the DeepNull DNN is not significantly affected by the choice of random seed. We use $\exp(x)$ and $\text{sigmoid}(x)$ as the non-linear function $f(\cdot)$ from Equation (9).

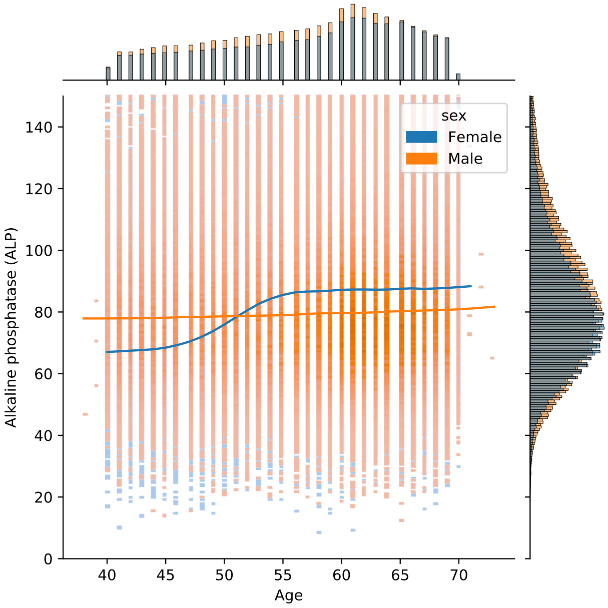

Supplementary Fig 4: **Alkaline phosphatase (ALP) distribution for each age in UKB.** The blue and orange lines are smoothed non-linear fits of ALP with respect to age.

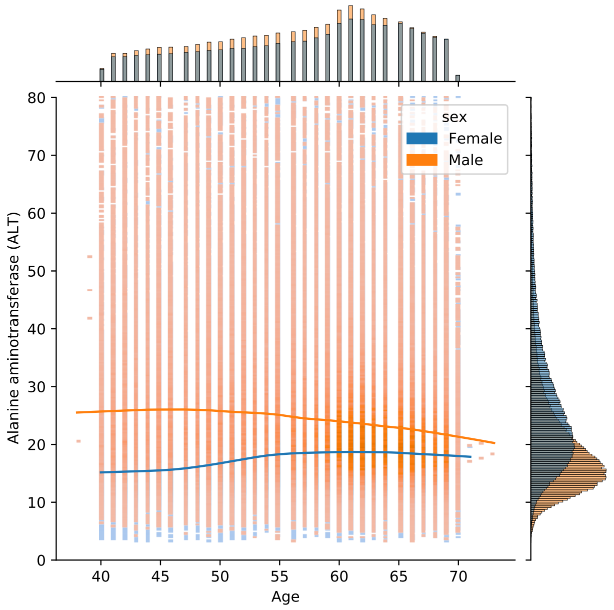

Supplementary Fig 5: **Alanine aminotransferase (ALT) distribution for each age in UKB.** The blue and orange lines are smoothed non-linear fits of ALT with respect to age.

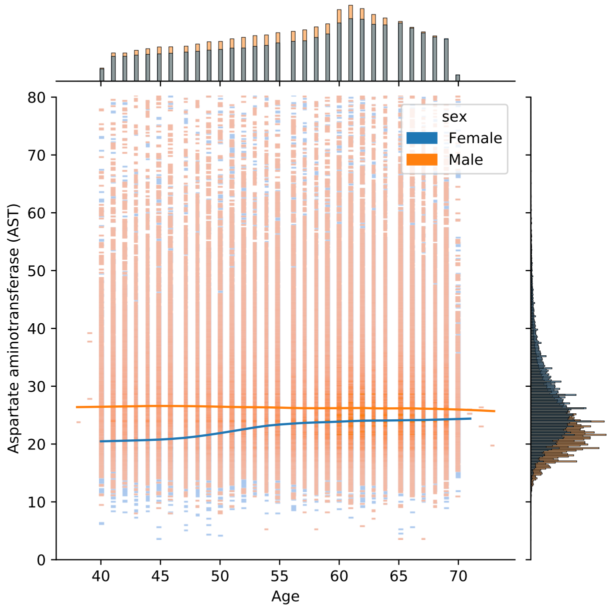

Supplementary Fig 6: **Aspartate aminotransferase (AST) distribution for each age in UKB.** The blue and orange lines are smoothed non-linear fits of AST with respect to age.

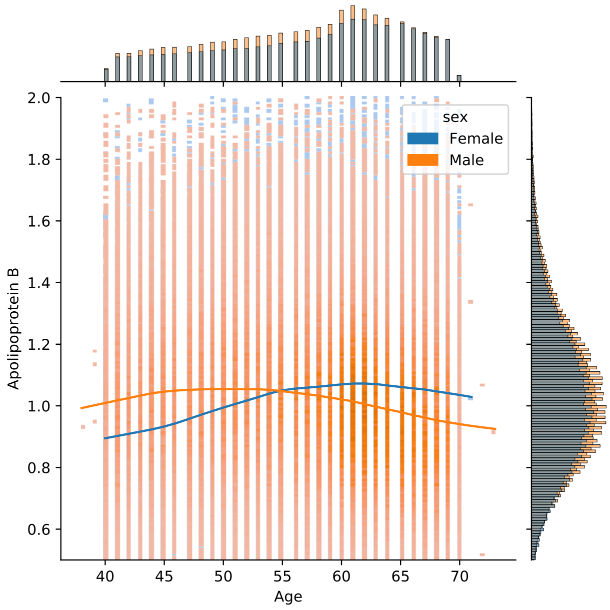

Supplementary Fig 7: **Apolipoprotein B (ApoB) distribution for each age in UKB.** The blue and orange lines are smoothed non-linear fits of ApoB with respect to age.

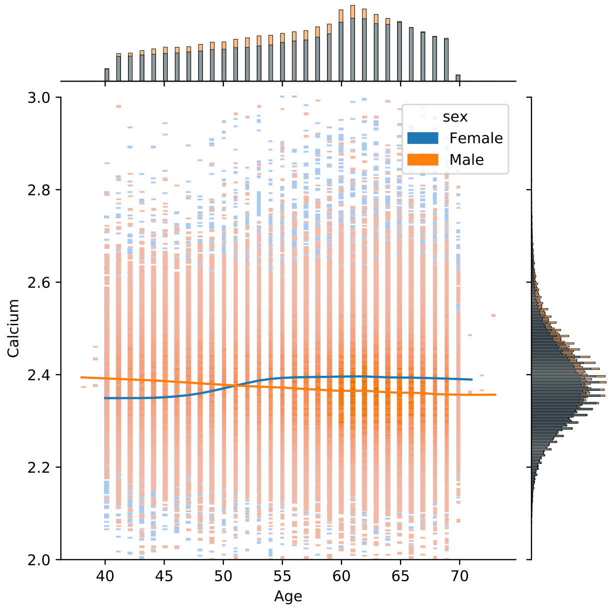

Supplementary Fig 8: **Calcium distribution for each age in UKB.** The blue and orange lines are smoothed non-linear fits of calcium with respect to age.

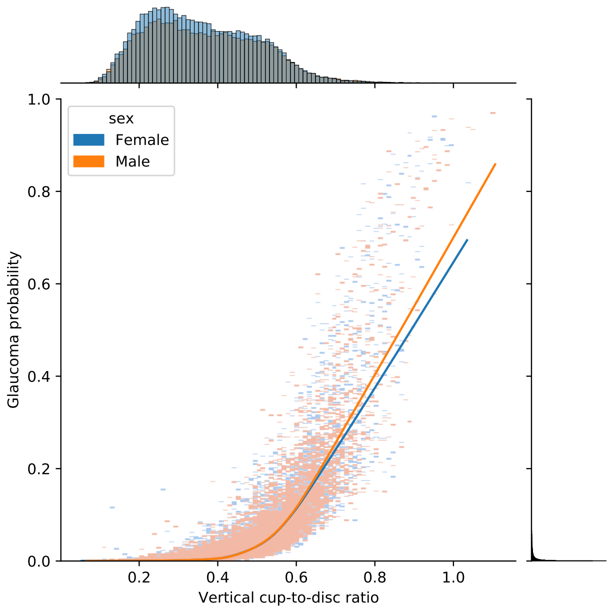

Supplementary Fig 9: **Glaucoma referral probability (GRP) distribution as a function of vertical cup-to-disc ratio (VCDR) in UKB.** The blue and orange lines are smoothed non-linear fits of GRP with respect to VCDR.

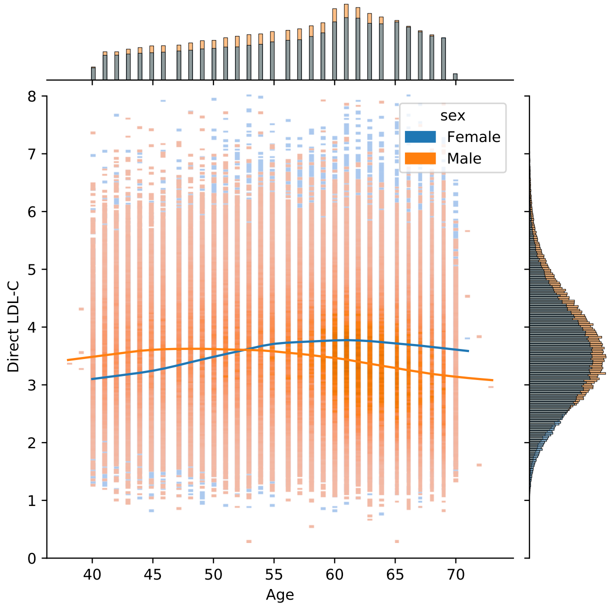

Supplementary Fig 10: **Low-density lipoprotein (LDL) distribution for each age in UKB.** The blue and orange lines are smoothed non-linear fits of LDL with respect to age.

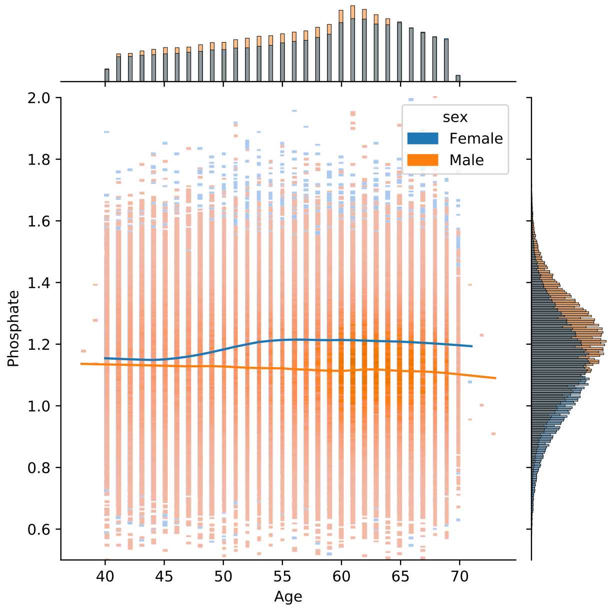

Supplementary Fig 11: **Phosphate distribution for each age in UKB.** The blue and orange lines are smoothed non-linear fits of Phosphate with respect to age.

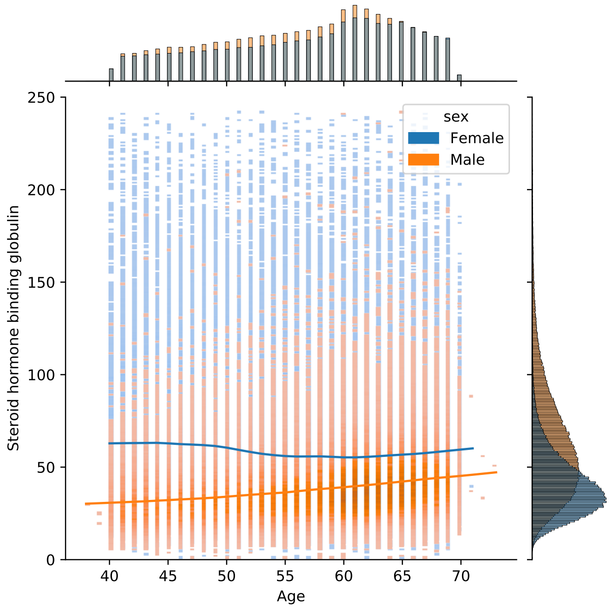

Supplementary Fig 12: **Sex hormone-binding globulin (SHBG) distribution for each age in UKB.** The blue and orange lines are smoothed non-linear fits of SHBG with respect to age.

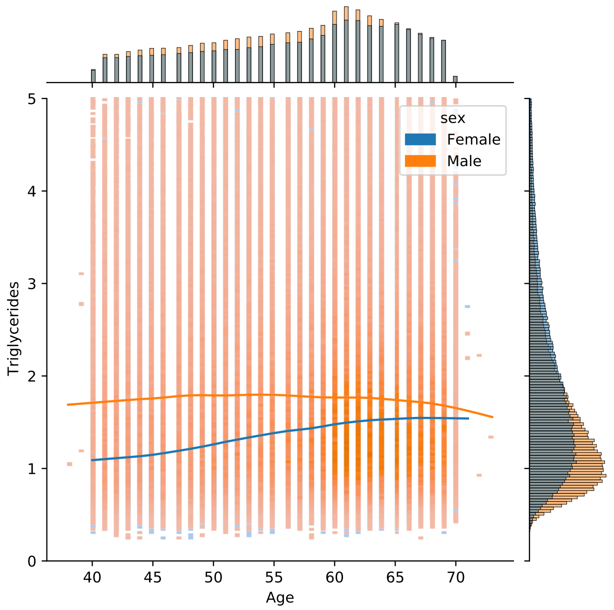

Supplementary Fig 13: **Triglycerides (TG) distribution for each age in UKB.** The blue and orange lines are smoothed non-linear fits of TG with respect to age.

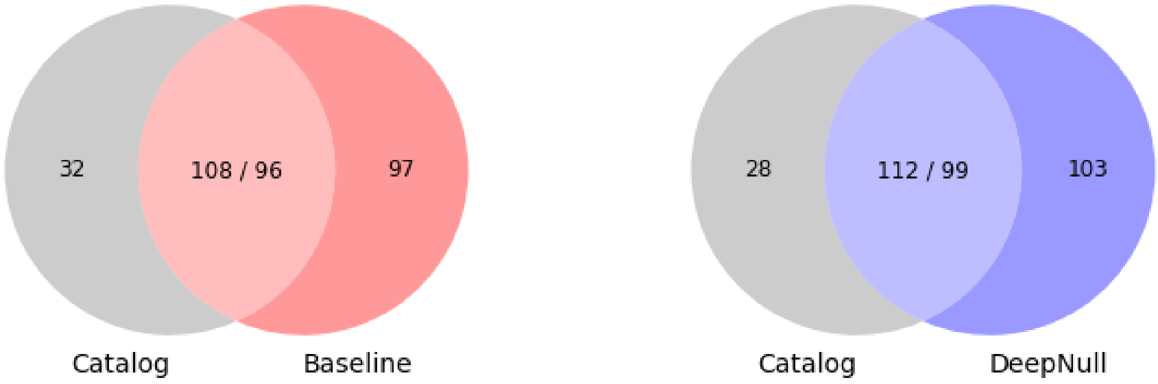

Supplementary Fig 14: **DeepNull identifies more loci associated with LDL than Baseline.** We computed the overlap of LDL loci for both DeepNull and Baseline with the GWAS catalog trait "LDL cholesterol". Numbers given in the Venn diagrams correspond to locus counts. Because locus overlap is not symmetric (a single GWAS catalog locus may overlap multiple Baseline or DeepNull loci, or vice versa), the shared section of each Venn diagram lists both the number of GWAS catalog loci and the number of Baseline or DeepNull loci.

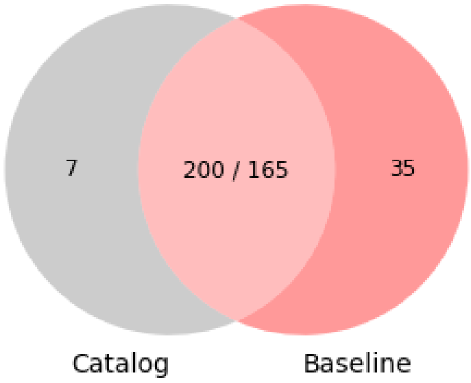

Supplementary Fig 15: **DeepNull identifies more loci associated with ApoB than Baseline.** We computed the overlap of ApoB loci for both DeepNull and Baseline with the GWAS catalog trait "Apolipoprotein B levels".

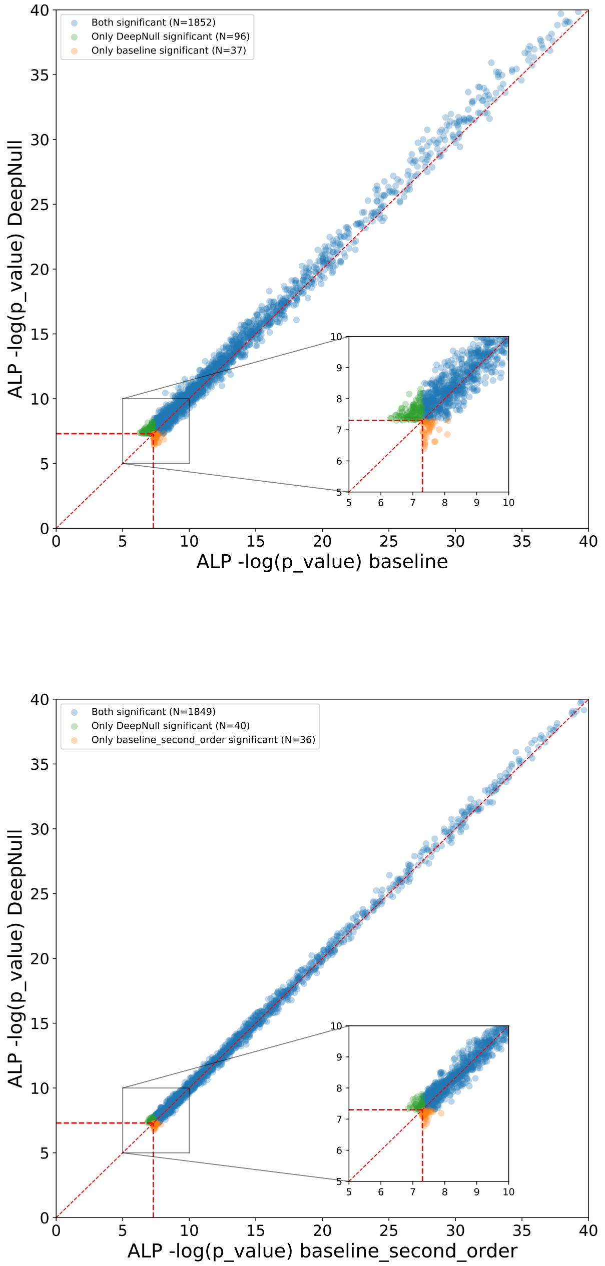

Supplementary Fig 16: **Significance level comparison of DeepNull vs Baseline and Second-order for Alkaline phosphatase (ALP).** The X-axis is the -log p-value of Baseline (top panel) and Second-order (bottom panel). The Y-axis is -log p-value of the DeepNull. Both Baseline and DeepNull p-values are computed using two-sided tests. The vertical and horizontal red line indicates the genome-wide significance level. The diagonal red line indicates the y=x. The orange dots indicate variants that are significant for Baseline/Second-order but not significant for DeepNull and green dots indicate variants that are significant for DeepNull but not significant for Baseline/Second-order. Plotted points represent "either-GWAS-significant variants". Briefly, variants from each GWAS are subset to independent genome-wide significant "hits" based on p-value and LD (Methods). To avoid biasing results toward either GWAS, all variants that are identified as hits in either GWAS are shown here. Note that multiple variants physically near each other, or in high LD, may both be plotted and consequently lead to different numbers of shared and uniquely significant variants than are reported in the hit and locus replication Supplementary Tables.

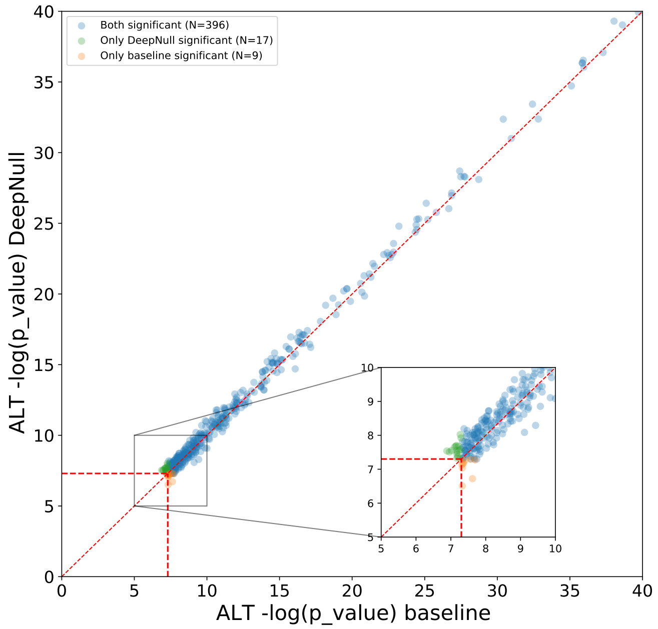

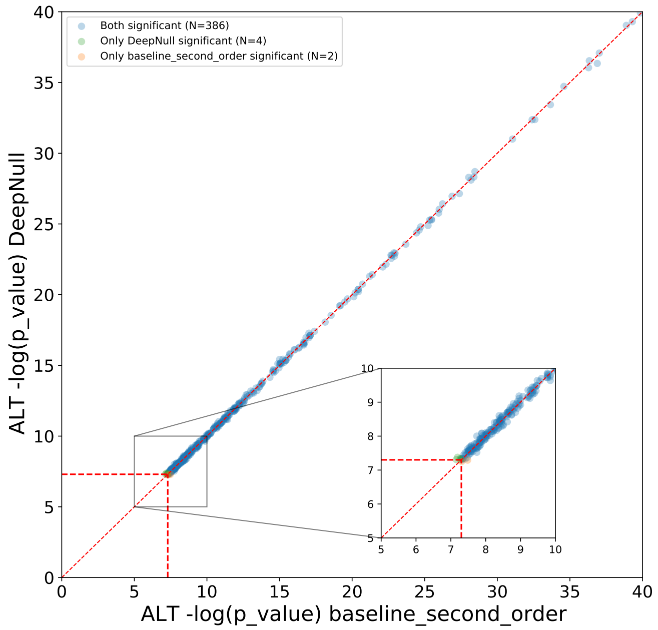

Supplementary Fig 17: **Significance level comparison of DeepNull vs Baseline and Second-order for Alanine aminotransferase (ALT).** The X-axis is the -log p-value of Baseline (top panel) and Second-order (bottom panel). The Y-axis is -log p-value of the DeepNull. The vertical and horizontal red line indicates the genome-wide significance level. The diagonal red line indicates the y=x. The orange dots indicate variants that are significant for Baseline/Second-order but not significant for DeepNull and green dots indicate variants that are significant for DeepNull but not significant for Baseline/Second-order.

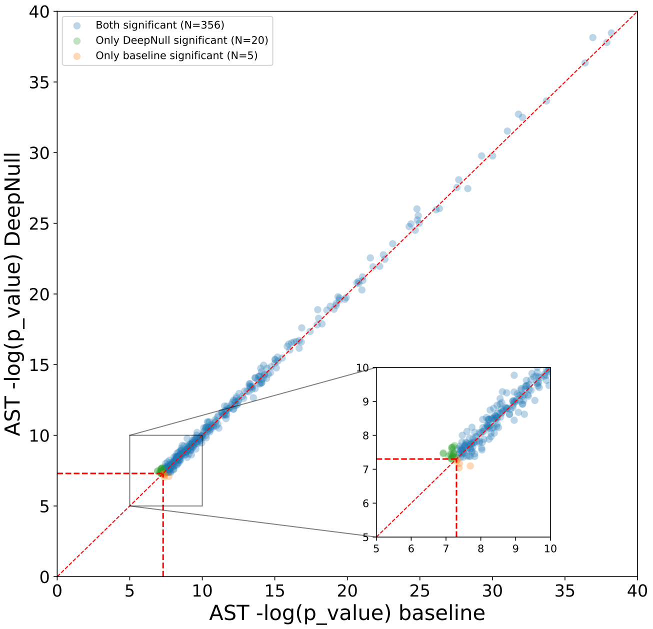

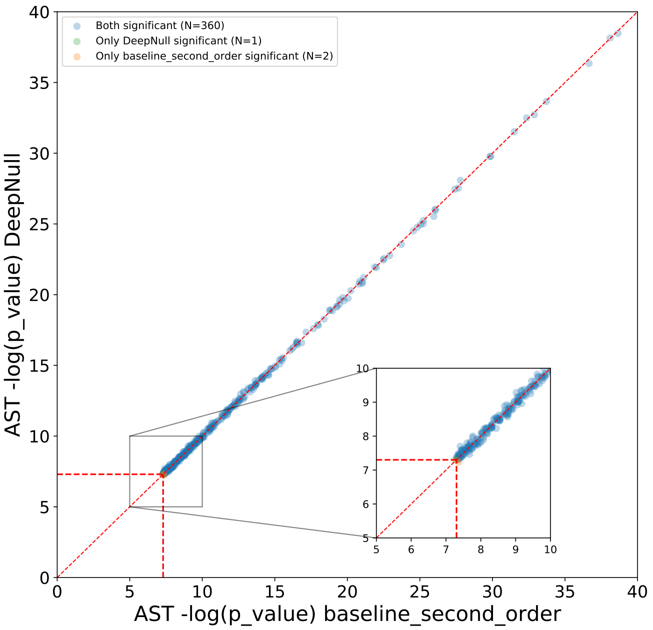

Supplementary Fig 18: **Significance level comparison of DeepNull vs Baseline and Second-order for Aspartate aminotransferase (AST).** The X-axis is the -log p-value of Baseline (left panel) and Second-order (right panel). The Y-axis is -log p-value of the DeepNull. The vertical and horizontal red line indicates the genome-wide significance level. The diagonal red line indicates the y=x. The orange dots indicate variants that are significant for Baseline/Second-order but not significant for DeepNull and green dots indicate variants that are significant for DeepNull but not significant for Baseline/Second-order.

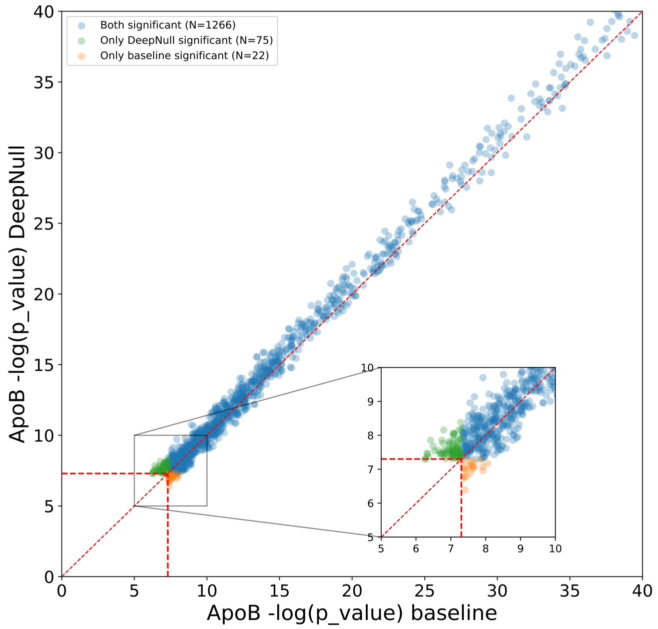

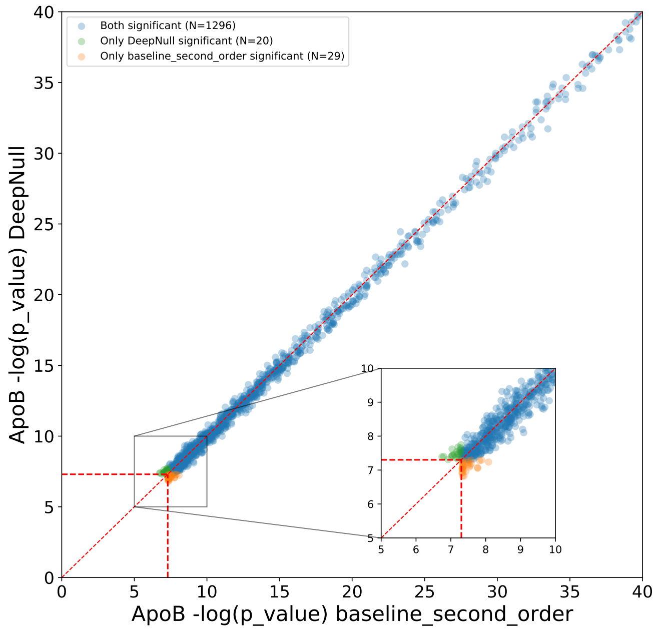

Supplementary Fig 19: **Significance level comparison of DeepNull vs Baseline and Second-order for Apolipoprotein B (ApoB).** The X-axis is the -log p-value of Baseline (top panel) and Second-order (bottom panel). The Y-axis is -log p-value of the DeepNull. The vertical and horizontal red line indicates the genome-wide significance level. The diagonal red line indicates the y=x. The orange dots indicate variants that are significant for Baseline/Second-order but not significant for DeepNull and green dots indicate variants that are significant for DeepNull but not significant for Baseline/Second-order.

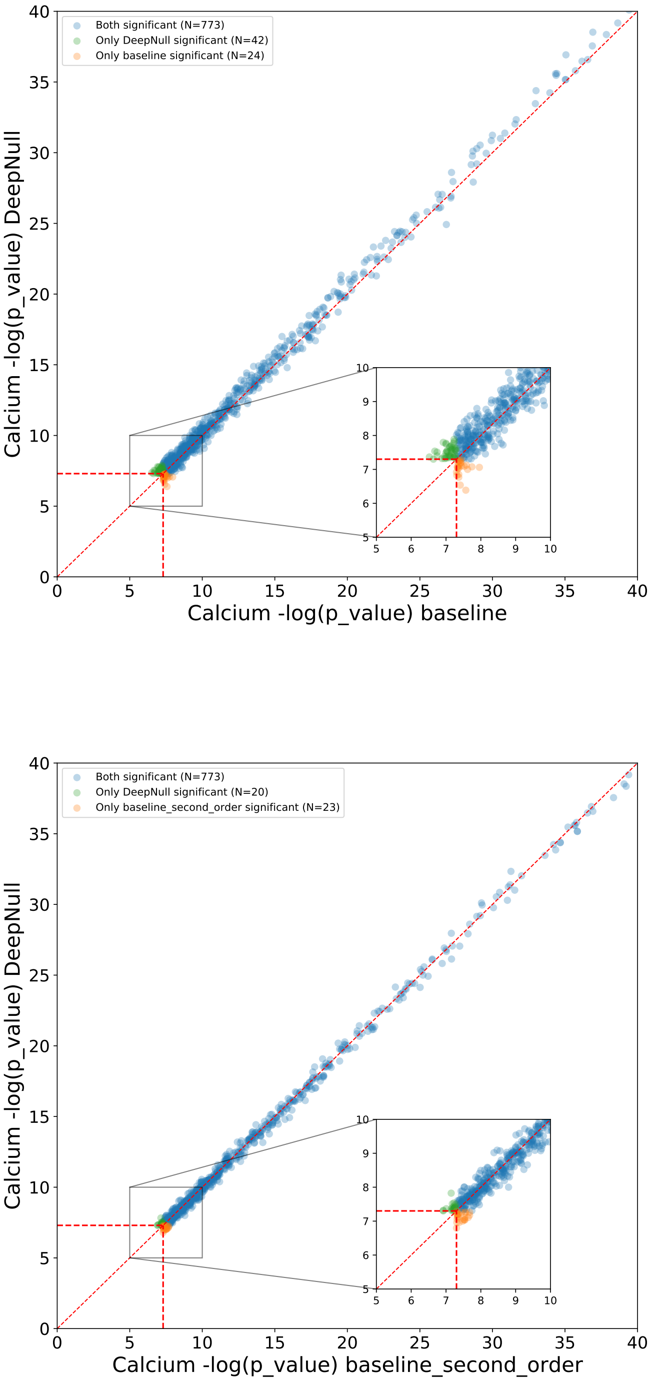

Supplementary Fig 20: **Significance level comparison of DeepNull vs Baseline and Second-order for Calcium.** The X-axis is the -log p-value of Baseline (top panel) and Second-order (bottom panel). The Y-axis is -log p-value of the DeepNull. The vertical and horizontal red line indicates the genome-wide significance level. The diagonal red line indicates the y=x. The orange dots indicate variants that are significant for Baseline/Second-order but not significant for DeepNull and green dots indicate variants that are significant for DeepNull but not significant for Baseline/Second-order.

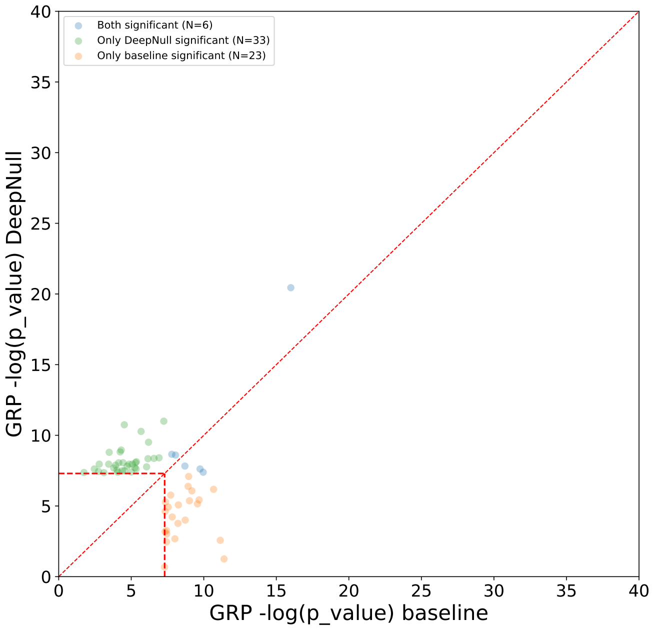

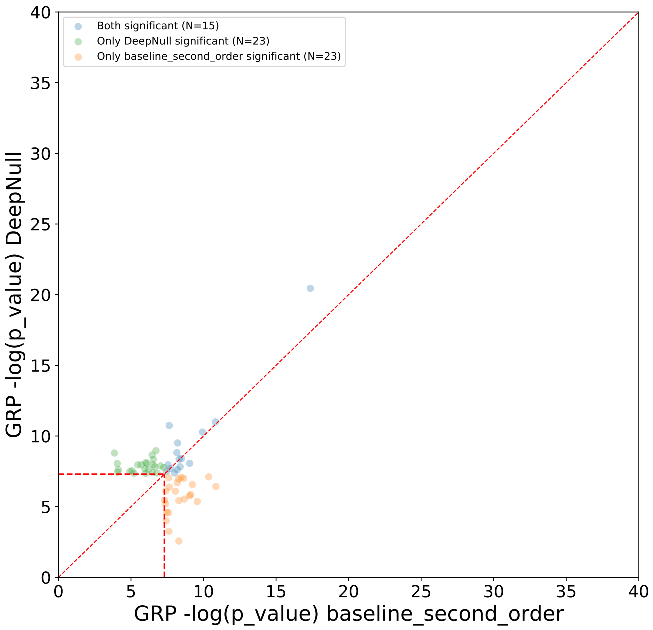

Supplementary Fig 21: **Significance level comparison of DeepNull vs Baseline and Second-order for Glaucoma referral probability (GRP).** The X-axis is the -log p-value of Baseline (top panel) and Second-order (bottom panel). The Y-axis is -log p-value of the DeepNull. The vertical and horizontal red line indicates the genome-wide significance level. The diagonal red line indicates the y=x. The orange dots indicate variants that are significant for Baseline/Second-order but not significant for DeepNull and green dots indicate variants that are significant for DeepNull but not significant for Baseline/Second-order.

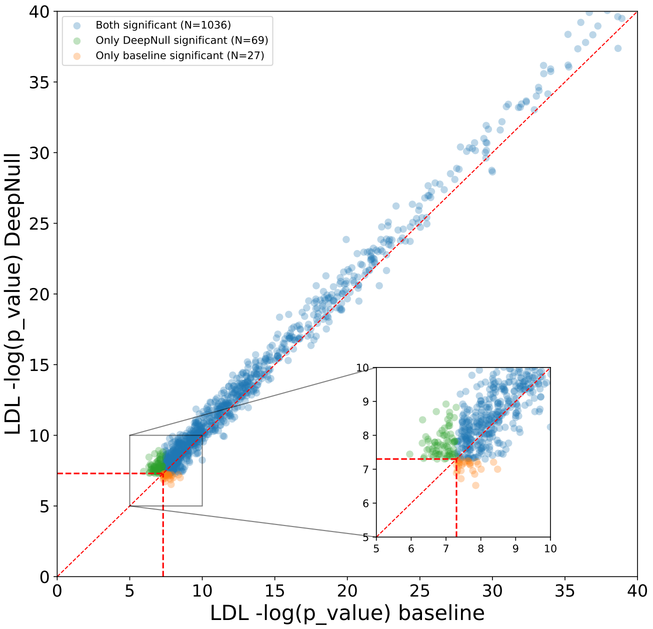

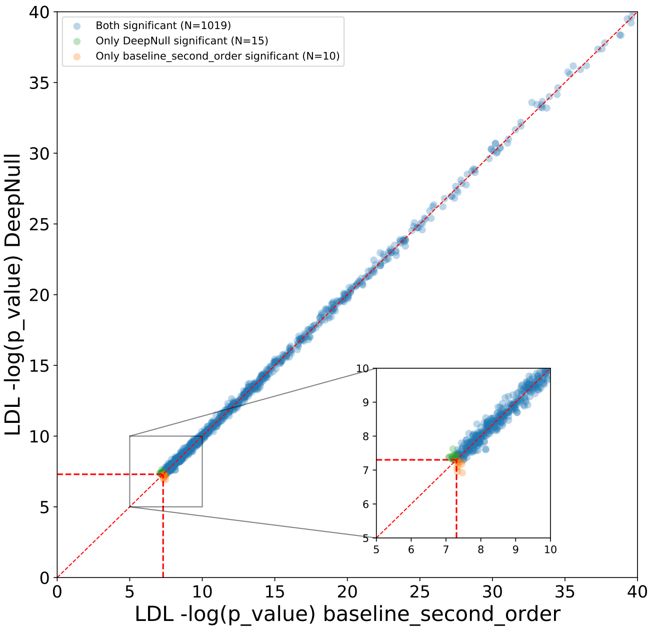

Supplementary Fig 22: **Significance level comparison of DeepNull vs Baseline and Second-order for Low-density lipoprotein (LDL).** The X-axis is the -log p-value of Baseline (top panel) and Second-order (bottom panel). The Y-axis is -log p-value of the DeepNull. The vertical and horizontal red line indicates the genome-wide significance level. The diagonal red line indicates the y=x. The orange dots indicate variants that are significant for Baseline/Second-order but not significant for DeepNull and green dots indicate variants that are significant for DeepNull but not significant for Baseline/Second-order.

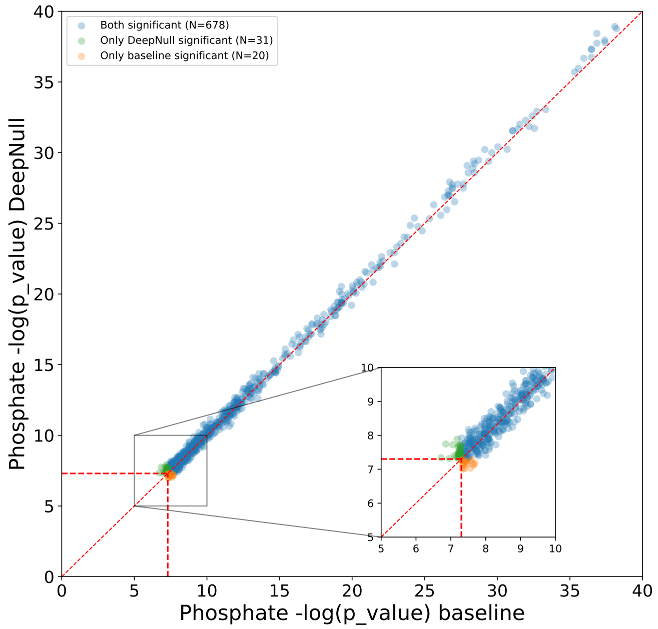

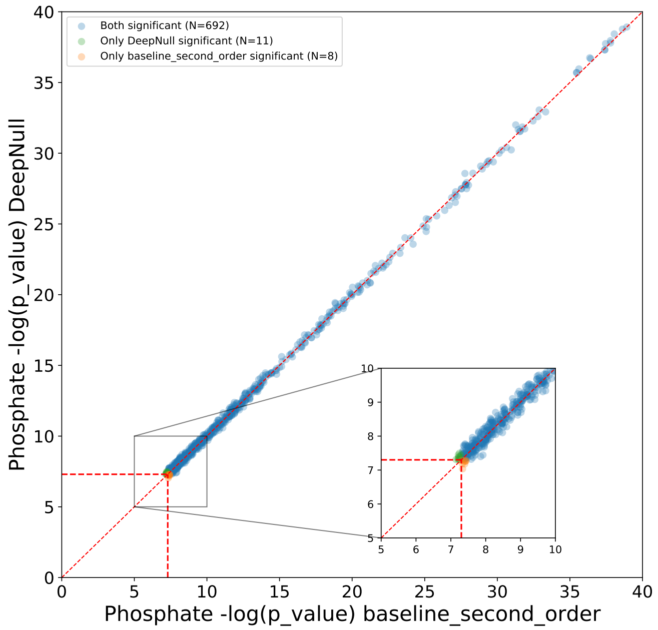

Supplementary Fig 23: **Significance level comparison of DeepNull vs Baseline and Second-order for Phosphate.** The X-axis is the -log p-value of Baseline (top panel) and Second-order (bottom panel). The Y-axis is -log p-value of the DeepNull. The vertical and horizontal red line indicates the genome-wide significance level. The diagonal red line indicates the y=x. The orange dots indicate variants that are significant for Baseline/Second-order but not significant for DeepNull and green dots indicate variants that are significant for DeepNull but not significant for Baseline/Second-order.

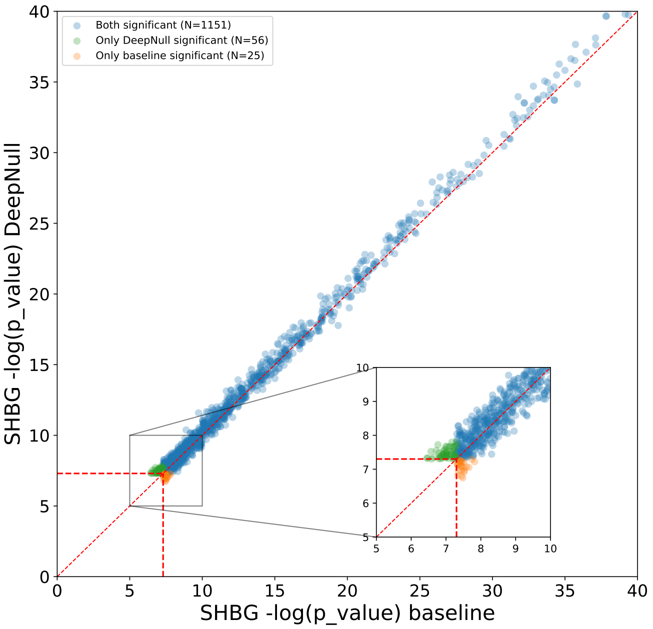

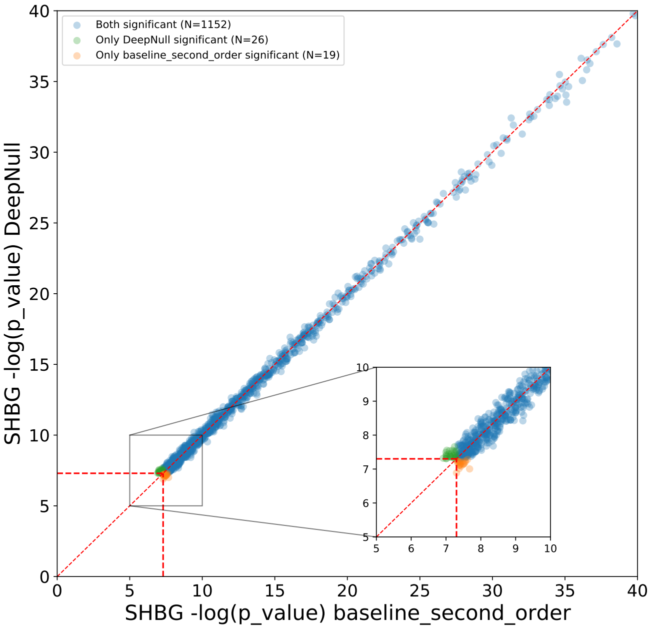

Supplementary Fig 24: **Significance level comparison of DeepNull vs Baseline and Second-order for Sex hormone-binding globulin (SHBG).** The X-axis is the -log p-value of Baseline (top panel) and Second-order (bottom panel). The Y-axis is -log p-value of the DeepNull. The vertical and horizontal red line indicates the genome-wide significance level. The diagonal red line indicates the y=x. The orange dots indicate variants that are significant for Baseline/Second-order but not significant for DeepNull and green dots indicate variants that are significant for DeepNull but not significant for Baseline/Second-order.

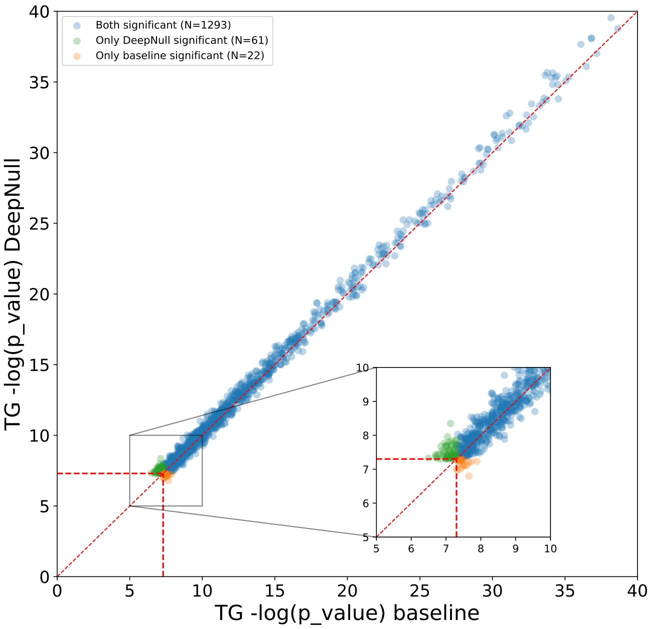

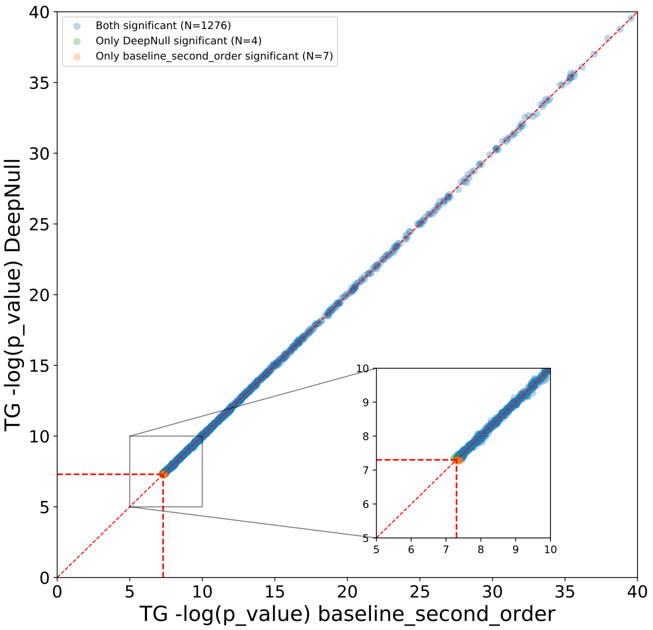

Supplementary Fig 25: **Significance level comparison of DeepNull vs Baseline and Second-order for Triglycerides (TG).** The X-axis is the -log p-value of Baseline (top panel) and Second-order (bottom panel). The Y-axis is -log p-value of the DeepNull. The vertical and horizontal red line indicates the genome-wide significance level. The diagonal red line indicates the y=x. The orange dots indicate variants that are significant for Baseline/Second-order but not significant for DeepNull and green dots indicate variants that are significant for DeepNull but not significant for Baseline/Second-order.

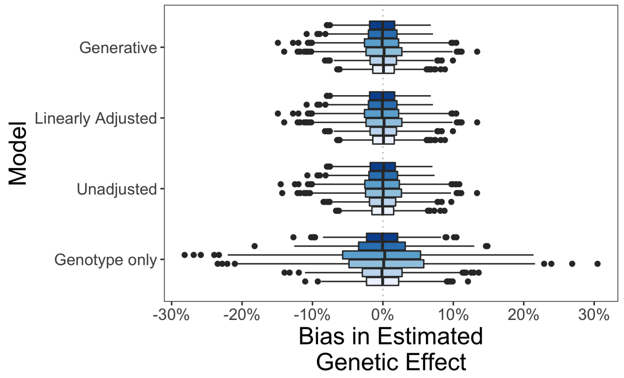

Supplementary Fig 26: **In the absence of dependence between genotype and the covariate, adjusting for the covariate is more efficient but not necessary for unbiased estimation.** Phenotypes were generated from the quadratic model. The genotype only model regresses y_i on $\bar{g}_i$. The unadjusted model regresses y_i on $\bar{g}_i$ and $h(x_i)$, where $h(x_i)$ is the prediction of y_i based on x_i. The linearly adjusted model regresses y_i on $h(x_i)$ and x_i. The generative model regresses y_i on x_i and x_i^2. Each box plot summarises results from $n = 10^3$ independent simulation replicates. The box demarcates, from left to right, the 25th, 50th, and 75th percentiles of the corresponding distribution. The whiskers extend between the largest and smallest values within 1.5 times the interquartile range. Any values outside the whiskers are marked by points.

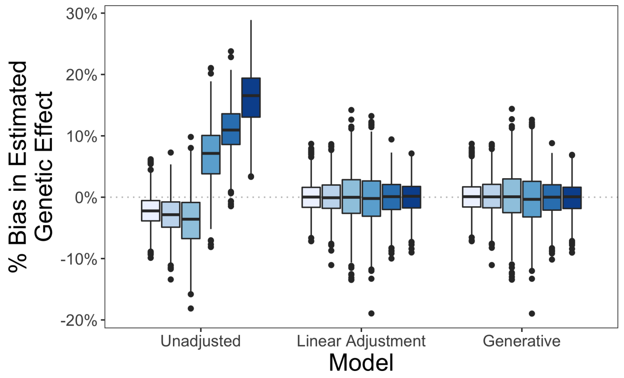

Supplementary Fig 27: **Linear adjustment provides unbiased estimation of the genetic effect in the case of a cubic phenotype, whereas the unadjusted model remains biased.** The unadjusted model regresses y_i on $\bar{g}_i$ and $h(x_i)$, where $h(x_i)$ is the prediction of y_i based on x_i. The linearly adjusted model regresses y_i on $h(x_i)$ and x_i. The generative model regresses y_i on x_i, x_i^2, and x_i^3. Each box plot summarises results from $n = 10^3$ independent simulation replicates. The box demarcates, from top to bottom, the 75th, 50th, and 25th percentiles of the corresponding distribution. The whiskers extend between the largest and smallest values within 1.5 times the interquartile range. Any values outside the whiskers are marked by points.

Supplementary Tables

Pheno	Linear Regression	Linear Regression 2-order interaction	Linear Regression 3-order interaction	Linear Regression 4-order interaction
ApoB	0.0951	0.2162	**0.2237**	0.1198
BMI	0.2114	0.2414	0.2561	**0.2734**

Supplementary Table 1: **The optimal number of covariate interactions for phenotype prediction depends on the phenotype and set of covariates.** We predicted BMI and ApoB phenotypes using `age`, `sex`, `genotyping_array`, 7 diet measures, 4 activity measures, and smoking status, in European individuals in UKB. We used linear regression to perform phenotype prediction with different numbers of covariate interactions. The 7 diet measures used were: 1) `Cooked vegetable intake` (Data-Field: 1289), 2) `Salad/raw vegetable intake` (Data-Field: 1299), 3) `Fresh fruit intake` (Data-Field: 1309), 4) `Bread intake` (Data-Field: 1438), 4) `Coffee intake` (Data-Field: 1498), 5) `Tea intake` (Data-Field: 1488), 6) `Water intake` (Data-Field: 1528), and 7) `Ever added salt to food` (Data-Field: 1478). We used the raw values for the diet measures (excluding the `Ever added salt to food`) and individuals with special values of -1, -3, and -10 were treated as missing. In the case of `Ever added salt to food`, we encoded "Never/rarely" as 0, "prefer not to answer" as missing, and all other values as 1. The 4 activity measures used were: 1) `Summed Metabolic Equivalent Task (MET) minutes per week for all activity` (Data-Field: 22040), 2) `Summed days activity` (Data-Field: 22033), 3) `Summed minutes for walking` (Data-Field: 22034), and 4) `Number of days/week of vigorous physical activity 10+ minutes` (Data-Field: 904). Table cells contain the Pearson correlation (R) between predicted and true phenotypic values. Bold values in the table indicate the best results.

σ_g^2	σ_x^2	Method	Power	$\mathbb{E}[\chi^2]$ Causal chr	Type I Error	$\mathbb{E}[\chi^2]$ Non-causal chr
0.2	0.1	DeepNull	0.2037 (0.0044)	1.8837 (0.0184)	0.0496 (0.0002)	1.0012 (0.0015)
		Baseline	0.2040 (0.0044)	1.8839 (0.0184)	0.0496 (0.0002)	1.0012 (0.0015)
0.2	0.2	DeepNull	0.2283 (0.0047)	1.9920 (0.0203)	0.0496 (0.0002)	1.0014 (0.0015)
		Baseline	0.2283 (0.0047)	1.9922 (0.0203)	0.0496 (0.0002)	1.0015 (0.0015)
0.4	0.1	DeepNull	0.3067 (0.0029)	2.6091 (0.0081)	0.0487 (0.0002)	0.9992 (0.0012)
		Baseline	0.3067 (0.0029)	2.6087 (0.0081)	0.0487 (0.0002)	0.9992 (0.0012)
0.4	0.2	DeepNull	0.3715 (0.0043)	2.9261 (0.0345)	0.0497 (0.0002)	1.0028 (0.0016)
		Baseline	0.3707 (0.0043)	2.9268 (0.0345)	0.0497 (0.0002)	1.0029 (0.0016)
0.4	0.4	DeepNull	0.3989 (0.0045)	3.4873 (0.0430)	0.0495 (0.0002)	1.0020 (0.0017)
		Baseline	0.3989 (0.0043)	3.4866 (0.0430)	0.0495 (0.0002)	1.0016 (0.0017)
0.6	0.2	DeepNull	0.3959 (0.0044)	3.7374 (0.0461)	0.0493 (0.0002)	1.0004 (0.0017)
		Baseline	0.3937 (0.0043)	3.7396 (0.0462)	0.0494 (0.0002)	1.0008 (0.0017)

Supplementary Table 2: **Comparison of Baseline and DeepNull models with only linear effects of covariates to phenotype.** Power is computed as the probability of detecting a variant as causal when the true simulated effect size is non-zero. $\mathbb{E}[\chi^2]$ is the expected χ^2 statistics for all variants. All values are computed by averaging over 100 simulated datasets and the values in parentheses are the standard error of the mean (s.e.m.) for each estimate. σ_g^2 is the phenotypic variance explained by genetic data (i.e. heritability). σ_x^2 is the phenotypic variance explained by covariates.

σ_g^2	σ_x^2	Method	Power	$\mathbb{E}[\chi^2]$ Causal chr	Type I Error	$\mathbb{E}[\chi^2]$ Non-causal chr
0.2	0.1	DeepNull	0.1962 (0.0047)	1.8581 (0.0171)	0.0499 (0.0002)	1.0035 (0.0014)
		Baseline	0.1962 (0.0047)	1.8581 (0.0171)	0.0499 (0.0002)	1.0036 (0.0014)
0.2	0.2	DeepNull	0.2146 (0.0049)	1.9298 (0.0195)	0.0499 (0.0002)	1.0038 (0.0014)
		Baseline	0.2147 (0.0049)	1.9300 (0.0196)	0.0499 (0.0002)	1.0038 (0.0014)
0.4	0.1	DeepNull	0.3437 (0.0048)	2.6795 (0.0305)	0.0502 (0.0002)	1.0061 (0.0015)
		Baseline	0.3436 (0.0048)	2.6794 (0.0306)	0.0501 (0.0002)	1.0060 (0.0015)
0.4	0.2	DeepNull	0.3571 (0.0049)	2.8112 (0.0350)	0.0501 (0.0002)	1.0063 (0.0015)
		Baseline	0.3565 (0.0049)	2.8117 (0.0350)	0.0501 (0.0002)	1.0064 (0.0015)
0.4	0.4	DeepNull	0.3778 (0.0059)	3.1678 (0.0514)	0.0499 (0.0002)	1.0062 (0.0016)
		Baseline	0.3789 (0.0057)	3.1685 (0.0513)	0.0499 (0.0002)	1.0061 (0.0016)
0.6	0.2	DeepNull	0.3995 (0.0046)	3.5943 (0.0482)	0.0499 (0.0002)	1.0059 (0.0021)
		Baseline	0.3984 (0.0048)	3.5941 (0.0482)	0.0499 (0.0002)	1.0056 (0.0021)

Supplementary Table 3: **Comparison of Baseline and DeepNull models with only linear effects of covariates to phenotype with missing non-confounding covariates.** We simulated phenotypes using a similar framework as Figure 1 where we used `age`, `sex`, and `genotype-array`. However, when we applied DeepNull and Baseline we provided only `sex` and `genotype-array`. Power is computed as the probability of detecting a variant as causal when the true simulated effect size is non-zero. $\mathbb{E}[\chi^2]$ is the expected χ^2 statistics for all variants. All values are computed by averaging over 100 simulated datasets and the values in parentheses are the standard error of the mean (s.e.m.) for each estimate. σ_g^2 is the phenotypic variance explained by genetic data (i.e. heritability). σ_x^2 is the phenotypic variance explained by covariates.

σ_g^2	σ_x^2	Method	Power	$\mathbb{E}[\chi^2]$ Causal chr	Type I Error	$\mathbb{E}[\chi^2]$ Non-causal chr
0.2	0.1	DeepNull	0.2051 (0.0045)	1.8871 (0.0173)	0.0499 (0.0002)	1.0038 (0.0014)
		Baseline	0.205 (0.0044)	1.8872 (0.0173)	0.0499 (0.0002)	1.0038 (0.0014)
0.2	0.2	DeepNull	0.2303 (0.0044)	1.9956 (0.0191)	0.0499 (0.0002)	1.0041 (0.0014)
		Baseline	0.2306 (0.0044)	1.9958 (0.0191)	0.0499 (0.0002)	1.0041 (0.0014)
0.4	0.1	DeepNull	0.3521 (0.0045)	2.7333 (0.0307)	0.0501 (0.0002)	1.0060 (0.0014)
		Baseline	0.3525 (0.0044)	2.7337 (0.0307)	0.0501 (0.0002)	1.0060 (0.0014)
0.4	0.2	DeepNull	0.3724 (0.0044)	2.9337 (0.0337)	0.0501 (0.0002)	1.0061 (0.0015)
		Baseline	0.3722 (0.0045)	2.9342 (0.0337)	0.0501 (0.0002)	1.0061 (0.0015)
0.4	0.4	DeepNull	0.3989 (0.0047)	3.4916 (0.0420)	0.0497 (0.0002)	1.0044 (0.0017)
		Baseline	0.3991 (0.0048)	3.4918 (0.0419)	0.0497 (0.0002)	1.0043 (0.0017)
0.6	0.2	DeepNull	3.7491 (0.0467)	3.5943 (0.0482)	0.0498 (0.0002)	1.0045 (0.0021)
		Baseline	0.3983 (0.0049)	3.7514 (0.0468)	0.0498 (0.0002)	1.0050 (0.0022)

Supplementary Table 4: **Comparison of Baseline and DeepNull models with only linear effects of covariates to phenotype with additional covariates with no effect on phenotypes.** We simulated phenotypes using a similar framework as Figure 1 where we used `sex` and `genotype-array`. However, when we applied DeepNull and Baseline we provided `age`, `sex`, and `genotype-array`. Power is computed as the probability of detecting a variant as causal when the true simulated effect size is non-zero. $\mathbb{E}[\chi^2]$ is the expected χ^2 statistics for all variants. All values are computed by averaging over 100 simulated datasets and the values in parentheses are the standard error of the mean (s.e.m.) for each estimate. σ_g^2 is the phenotypic variance explained by genetic data (i.e. heritability). σ_x^2 is the phenotypic variance explained by covariates.

σ_g^2	σ_x^2	Method	Power	$\mathbb{E}[\chi^2]$ Causal chr	Type I Error	$\mathbb{E}[\chi^2]$ Non-causal chr
0.2	0.1	DeepNull	**0.2061 (0.0045)**	**2.0671 (0.0195)**	0.0495 (0.0002)	1.0004 (0.0015)
		Baseline	0.1956 (0.0046)	2.0169 (0.0195)	0.0495 (0.0002)	1.0007 (0.0015)
		Relative %	5.64	2.52		
0.2	0.2	DeepNull	**0.2334 (0.0047)**	**2.2000 (0.0215)**	0.0495 (0.0002)	1.0006 (0.001)
		Baseline	0.2062 (0.0048)	2.0755 (0.0224)	0.0495 (0.0002)	1.0010 (0.001)
		Relative %	13.1	6.02		
0.4	0.1	DeepNull	**0.3645 (0.0051)**	**3.1081 (0.0339)**	0.0499 (0.0002)	1.0023 (0.0016)
		Baseline	0.3557 (0.0052)	3.0078 (0.0347)	0.0498 (0.0002)	1.0027 (0.0016)
		Relative %	2.47	3.33		
0.4	0.2	DeepNull	**0.3843 (0.0049)**	**3.3576 (0.0371)**	0.0499 (0.0002)	1.0027 (0.0016)
		Baseline	0.3606 (0.0054)	3.1165 (0.0403)	0.0499 (0.0002)	1.0031 (0.0017)
		Relative %	6.7	7.9		
0.4	0.4	DeepNull	**0.4013 (0.0044)**	**4.0642 (0.0450)**	0.0501 (0.0002)	1.0038 (0.0018)
		Baseline	0.3740 (0.0063)	3.3896 (0.0574)	0.0497 (0.0002)	1.0028 (0.0017)
		Relative %	7.29	19.90		
0.6	0.2	DeepNull	0.3973 (0.0043)	**4.4031 (0.0499)**	0.0505 (0.0003)	1.0072 (0.0027)
		Baseline	0.3906 (0.0043)	4.0631 (0.0536)	0.0498 (0.0002)	1.0031 (0.0018)
		Relative %	-	8.36		

Supplementary Table 5: **Comparison of Baseline and DeepNull models with covariate interactions.** This result is obtained from a similar process as Figure 2. All values are computed by averaging over 100 simulated datasets and the values in parentheses are the standard error of the mean (s.e.m.) for each estimate. Bold values in the table indicate the best results.

$f(\cdot)$	Method	Power	$\mathbb{E}[\chi^2]$ Causal chr	Type I Error	$\mathbb{E}[\chi^2]$ Non-causal chr
	DeepNull	**0.4028 (0.0048)**	**4.0520 (0.0451)**	0.0500 (0.0002)	1.0030 (0.0018)
$\sin(x)$	Baseline	0.3893 (0.0058)	3.5788 (0.0540)	0.0498 (0.0002)	1.0041 (0.0018)
	Relative %	3.45	13.22		
	DeepNull	**0.4044 (0.0046)**	**4.0026 (0.0443)**	0.0500 (0.0002)	1.0024 (0.0017)
$\sin(20x)$	Baseline	0.3756 (0.0061)	3.3506 (0.0551)	0.0501 (0.0002)	1.0042 (0.0016)
	Relative %	7.73	19.42		
	DeepNull	**0.4022 (0.0041)**	**4.0542 (0.0451)**	0.0500 (0.0002)	1.0035 (0.0017)
$\exp(x)$	Baseline	0.3839 (0.0058)	3.4504 (0.0575)	0.0498 (0.0002)	1.0039 (0.0017)
	Relative %	5.74	17.52		
	DeepNull	**0.4031 (0.0046)**	**4.0324 (0.0455)**	0.0502 (0.0002)	1.0040 (0.0019)
$\exp(20x)$	Baseline	0.3903 (0.0057)	3.2981 (0.0557)	0.0500 (0.0002)	1.0030 (0.0017)
	Relative %	4.35	22.2		
	DeepNull	**0.4002 (0.0043)**	**4.0597 (0.0452)**	0.0502 (0.0002)	1.0051 (0.0020)
$\log(\|x\|)$	Baseline	0.3720 (0.0065)	3.488 (0.0679)	0.0495 (0.0002)	1.0011 (0.0017)
	Relative %	8.35	16.37		
	DeepNull	**0.4045 (0.0043)**	**4.0598 (0.0453)**	0.05017 (0.0002)	1.0046 (0.0018)
$\log(\|20x\|)$	Baseline	0.3720 (0.0065)	3.4888 (0.0679)	0.0495 (0.0002)	1.0011 (0.0017)
	Relative %	8.89	16.3		
	DeepNull	**0.4024 (0.0043)**	**4.0615 (0.0451)**	0.0501 (0.0002)	1.003 (0.0017)
sigmoid(x)	Baseline	0.3751 (0.0062)	3.3932 (0.0572)	0.0498 (0.0002)	1.003 (0.0016)
	Relative %	7.60	19.68		
	DeepNull	**0.4000 (0.0041)**	**4.0526 (0.0450)**	0.0500 (0.0002)	1.0031 (0.0018)
sigmoid($20x$)	Baseline	0.3848 (0.0056)	3.4897 (0.0540)	0.0500 (0.0002)	1.0048 (0.0018)
	Relative %	4.16	16.13		

Supplementary Table 6: **Comparison of Baseline and DeepNull models under a simulation of a non-linear effect of the covariates on the phenotype.** We considered the genetic architecture where we set $\sigma_g^2 = 0.4$ and $\sigma_x^2 = 0.4$. This result is obtained from similar process as Table 2 while using `age`, `sex`, `genotyping_array`, `age`^{2}, `age` $\times$ `sex`, `age` $\times$ `genotyping_array` as true covariates to simulate datasets, however, both Baseline and DeepNull use `age`, `sex`, and `genotype_array` as input covariates. All values are averaged over 100 simulations and the parenthetical values are the standard errors of the mean for each estimate. Bold values in the table indicate the best results.

$f(\cdot)$	n	Power	$\mathbb{E}[\chi^2]$ Causal chr	Type I error	$\mathbb{E}[\chi^2]$ Non-causal chr
Exp	20,000	0.5425 (0.0052)	5.7107 (0.0604)	0.0497 (0.0003)	0.9999 (0.0024)
Exp	50,000	0.7202 (0.0053)	11.2885 (0.1828)	0.0543 (0.0011)	1.0413 (0.0096)
Exp	100,000	0.7982 (0.0040)	25.6248 (0.3505)	0.0518 (0.0007)	1.0210 (0.0065)
Exp	200,000	0.8683 (0.0042)	41.3273 (0.6022)	0.0495 (0.0002)	1.0013 (0.0019)
Sigmoid	20,000	0.5414 (0.0051)	5.7199 (0.0609)	0.0497 (0.0003)	1.0004 (0.0024)
Sigmoid	50,000	0.7173 (0.0054)	11.2115 (0.1757)	0.0534 (0.0010)	1.0337 (0.0084
Sigmoid	100,000	0.7976 (0.0039)	25.5486 (0.3420)	0.0516 (0.0007)	1.0179 (0.0060)
Sigmoid	200,000	0.8684 (0.0042)	41.2968 (0.6049)	0.0494 (0.0002)	1.0002 (0.0019)

Supplementary Table 7: **DeepNull results improve as sample size increases.** We computed the power and expected χ^2 statistics of the causal chromosome, type I error, and the expected χ^2 statistics of the non-causal chromosomes. We considered the genetic architecture where we set $\sigma_g^2 = 0.4$ and $\sigma_x^2 = 0.4$ and then simulated phenotypes for different number of individuals (n) using UKB genotype and covariates. We considered two non-linear functions of $\exp(x)$ and sigmoid(x) for $f(\cdot)$ in Equation (9). All values are computed by averaging over 100 simulated datasets and the values in parentheses are the standard error of the mean (s.e.m.) for each estimate.

Pheno	MSE	MAE
ALP	658.900	17.458
ALT	180.600	8.210
AST	106.725	5.535
Apob	0.054	0.185
Calcium	0.008	0.071
GRP	0.001	0.013
LDL	0.717	0.672
Phosphate	0.023	0.122
SHBG	631.372	18.167
Triglycerides	0.980	0.713

Supplementary Table 8: **DeepNull's prediction performance.** We compute the mean squared error (MSE) and mean absolute error (MAE) of DeepNull's prediction for all 10 UKB phenotypes.

Pheno	Fold 0	Fold 1	Fold 2	Fold 3	Fold 4
AST	0.1892	0.2156	0.2055	0.1969	0.1998
ApoB	0.1821	0.1866	0.1898	0.1927	0.1926
GRP	0.8487	0.8505	0.8426	0.8603	0.8626
LDL	0.2141	0.2174	0.2215	0.2242	0.2236

Supplementary Table 9: **DeepNull's prediction accuracy (R) by fold.** Here we present the fold-level prediction accuracy for the four phenotypes with largest evidence of non-linear interactions: AST, ApoB, GRP, and LDL. The open-source DeepNull software performs this check by default.

Pheno	S-LDSC Intercept		S-LDSC SNP-heritability	
	Baseline	DeepNull	Baseline	DeepNull
ALP	1.0722 (0.0713)	1.0751 (0.0733)	0.1901 (0.0192)	0.1945 (0.0196)
ALS	1.0440 (0.0222)	1.0452 (0.0225)	0.0853 (0.0065)	0.0865 (0.0066)
AST	1.0345 (0.0194)	1.0352 (0.0193)	0.0606 (0.0054)	0.0610 (0.0054)
ApoB	1.0724 (0.0291)	1.0762 (0.0298)	0.1277 (0.0131)	0.1312 (0.0131)
Calcium	1.0962 (0.0309)	1.0979 (0.0315)	0.1260 (0.0077)	0.1281 (0.0079)
GRP	0.9991 (0.0086)	1.0044 (0.0089)	0.0237 (0.0113)	0.0221 (0.0123)
LDL	1.0540 (0.0262)	1.0575 (0.0267)	0.1120 (0.0109)	0.1161 (0.0113)
Phosphate	1.0423 (0.0242)	1.0432 (0.0244)	0.1312 (0.0088)	0.1327 (0.0089)
SHBG	1.1677 (0.0799)	1.1724 (0.0807)	0.1533 (0.0138)	0.1559 (0.0139)
TG	1.1133 (0.04)	1.1158 (0.0406)	0.1553 (0.0127)	0.1572 (0.0129)

Supplementary Table 10: **S-LDSC results on Baseline and DeepNull.** We computed the S-LDSC intercept and SNP-heritability. In all phenotypes the S-LDSC intercept is the same for both Baseline and DeepNull which indicates no confounding. Values in parentheses are the standard error of the mean (s.e.m) obtained from S-LDSC.

	Baseline		DeepNull	
Pheno	#Sets	Avg. $-\log(p)$	#Sets	Avg. $-\log(p)$
ApoB	983	7.65	946	7.81
GRP	0	$--$	42	4.86
LDL	955	8.60	1000	8.38

Supplementary Table 11: **FUMA Gene Set Enrichment Analysis.** Comparison of Baseline and DeepNull with respect to the number of significantly enriched gene sets (#Sets) and the average $-\log_{10}$(p-value) of the significant gene sets. Enrichment p-values are based on one-sided hypergeometric tests.

Pheno	#Hits			#Loci		
	Baseline	Baseline + ReLU	DeepNull	Baseline	Baseline + ReLU	DeepNull
ALP	1697	1705	**1759**	336	335	**350**
ALT	371	375	**379**	173	172	**174**
AST	337	341	**351**	137	140	**145**
ApoB	1172	1192	**1219**	200	209	**217**
Calcium	726	732	**739**	272	275	**281**
GRP	28	**38**	**38**	26	36	**38**
LDL	950	956	**993**	193	198	**202**
Phosphate	658	**667**	664	224	**230**	229
SHBG	1084	1081	**1120**	319	316	**323**
TG	1221	1229	**1254**	261	264	**266**

Supplementary Table 12: **DeepNull consistently detects more hits and loci compared to Baseline and Baseline+ReLU.** Baseline+ReLU is a model where we add additional covariates to Baseline model. We obtained these additional covariates by applying the ReLU function on the covariate of interest using 5 different thresholds. For all phenotypes excluding the GRP, we add 5 additional ReLU based on age. In the case of GRP, we used 10 additional ReLU where 5 covariates are computed using age and another 5 are computed using VCDR.

Pheno	#Hits	#Loci	R^2	S-LDSC Intercept	S-LDSC SNP-heritability
ALP	1750	343	0.1509 (0.1464, 0.1560)	1.0730 (0.0727)	0.1934 (0.0195)
ALT	377	173	0.1121 (0.1089, 0.1152)	1.0456 (0.0226)	0.0864 (0.0066)
AST	351	145	0.0643 (0.0615, 0.0673)	1.0352 (0.0193)	0.0611 (0.0054)
ApoB	1231	211	0.1491 (0.1470, 0.1508)	1.0816 (0.0297)	0.1305 (0.0129)
Calcium	743	280	0.0882 (0.0862, 0.0897)	1.0981 (0.0315)	0.1286 (0.008)
GRP	38	35	0.6492 (0.6371, 0.6595)	0.9907 (0.0091)	0.0372 (0.0124)
LDL	991	200	0.1337 (0.1318, 0.1358)	1.0598 (0.0268)	0.1162 (0.0113)
Phosphate	663	226	0.1189 (0.1171, 0.1207)	1.0457 (0.0243)	0.1318 (0.0089)
SHBG	1113	319	0.2624 (0.2596, 0.2644)	1.1738 (0.0809)	0.1565 (0.0140)
TG	1256	267	0.1440 (0.1419, 0.1461)	1.1153 (0.0407)	0.1575 (0.0129)

Supplementary Table 13: **Baseline model with additional second order interaction covariates.** We ran Baseline method with additional covariates that include $\texttt{age}^2$, $\texttt{age} \times \texttt{sex}$, $\texttt{age} \times \texttt{genotyping\_array}$, and $\texttt{sex} \times \texttt{genotyping\_array}$. These covariates supplement the top 15 PCs, `age`, `sex`, and `genotyping_array` included in the Baseline model. R^2 is measurement of the phenotype prediction where R is the Pearson correlation between true phenotype and predicted values. The phenotype prediction is the combination of PRS computed using the PLINK `--score` method and linear effects of covariates to phenotype. Here, we show the 95% confidence intervals for the squared Pearson's correlation measuring the correlation between the models predicted value and the true values. We compute the S-LDSC intercept and SNP-heritability. We observe that in all four phenotypes the S-LDSC intercept is the same for both Baseline and DeepNull which indicates no confounding. Values in parentheses for S-LDSC intercept and SNP-heritability are the standard error of the mean (s.e.m).

Pheno	Baseline vs DeepNull			Second-order vs DeepNull		
	Baseline-only	Shared	DeepNull-only	Second-only	Shared	DeepNull-only
ALP	8	1737	22	7	1749	10
ALT	5	376	3	1	375	4
AST	1	341	10	0	351	0
ApoB	4	1196	23	4	1211	8
Calcium	8	719	20	6	733	6
GRP	18	8	30	19	16	22
LDL	6	976	17	2	989	4
Phosphate	8	651	13	2	660	4
SHBG	10	1106	14	5	1111	9
TG	6	1239	15	2	1252	2

Supplementary Table 14: **Number of significant hits shared between DeepNull and Baseline and Second-order Baseline.** In the case of Baseline, we performed GWAS analysis while including `age`, `sex`, `genotyping_array`, and top 15 PCs as possible covariates. In the case of Second-order (2-order) Baseline, we ran Baseline model with additional covariates that includes $\texttt{age}^2$, `age` × `sex`, `age` × `genotyping_array`, and `sex` × `genotyping_array`. These covariates supplement the top 15 PCs, `age`, `sex`, and `genotyping_array` included in the Baseline model. "-only" indicates hits that are detected only by one the methods while the "Shared" indicates DeepNull hits that overlap one or more hits from the baseline method. Note that shared hits are not symmetric, but the qualitative results are the same as if we reported the shared baseline method hits.

Pheno	Baseline vs DeepNull			Second-order vs DeepNull		
	Baseline-only	Shared	DeepNull-only	Second-only	Shared	DeepNull-only
ALP	4	333	17	4	340	10
ALT	3	171	3	1	172	2
AST	1	136	9	0	145	0
ApoB	1	199	18	2	210	7
Calcium	8	262	19	6	275	6
GRP	18	8	30	19	16	22
LDL	5	188	14	2	198	4
Phosphate	8	217	12	2	225	4
SHBG	7	310	13	5	314	9
TG	6	254	12	1	265	1

Supplementary Table 15: **Number of significant loci shared between DeepNull and Baseline and Second-order Baseline.** This analysis is similar to Supplementary Table 14; however, here we report the number of loci instead of hits.

Pheno	Spline vs DeepNull			XGBoost vs DeepNull		
	Spline-only	Shared	DeepNull-only	XGBoost-only	Shared	DeepNull-only
ALP	8	1736	23	1	1758	1
ALT	3	375	4	1	377	2
AST	1	347	4	0	351	0
ApoB	5	1204	15	4	1212	7
Calcium	2	727	12	9	734	5
GRP	20	13	25	11	26	12
LDL	4	987	6	5	992	1
Phosphate	7	658	6	5	660	4
SHBG	11	1105	15	8	1115	5
TG	6	1246	8	5	1250	4

Supplementary Table 16: **Number of significant hits shared between DeepNull and Spline and XGBoost Baseline.**

Pheno	Spline vs DeepNull			XGBoost vs DeepNull		
	Spline-only	Shared	DeepNull-only	XGBoost-only	Shared	DeepNull-only
ALP	4	332	18	1	349	1
ALT	2	172	2	0	173	1
AST	1	142	3	0	145	0
ApoB	2	204	13	2	211	6
Calcium	2	270	11	8	276	5
GRP	20	13	25	11	26	12
LDL	4	197	5	5	201	1
Phosphate	6	224	5	5	225	4
SHBG	8	309	14	6	318	5
TG	5	261	5	3	264	2

Supplementary Table 17: **Number of significant loci shared between DeepNull and Spline and XGBoost Baseline.** This analysis is similar to Supplementary Table 16; however, here we report the number of loci instead of hits.

Pheno	Baseline	DeepNull-Baseline	DeepNull	% Δ (P)
ALP	0.1353 (0.1312, 0.1397)	0.1363 (0.1323, 0.1406)	0.1569 (0.1523, 0.1623)	16.01 (3.93×10^{-52})
ALT	0.0970 (0.0940, 0.0997)	0.0970 (0.0940, 0.0997)	0.1127 (0.1094, 0.1157)	16.16 (2.84×10^{-35})
AST	0.0566 (0.0541, 0.0595)	0.0574 (0.0549, 0.0602)	0.0642 (0.0616, 0.0672)	13.32 (5.07×10^{-14})
ApoB	0.1159 (0.1142, 0.1173)	0.1166 (0.1149, 0.1181)	0.1410 (0.1390, 0.1424)	21.61 (6.01×10^{-75})
Calcium	0.0682 (0.0666, 0.0697)	0.0690 (0.0674, 0.0706)	0.0845 (0.0827, 0.0860)	23.90 (1.22×10^{-44})
GRP	0.3958 (0.3889, 0.4022)	0.3950 (0.3872, 0.4011)	0.7259 (0.7124, 0.7366)	83.42 ($\leq 1.00 \times 10^{-200}$)
LDL	0.0950 (0.0935, 0.0967)	0.0956 (0.0940, 0.0973)	0.1334 (0.1315, 0.1352)	40.33 (6.56×10^{-189})
SHBG	0.2454 (0.2428, 0.2475)	0.2450 (0.2425, 0.2471)	0.2581 (0.2555, 0.2602)	5.19 (7.74×10^{-14})
Phosphate	0.1111 (0.1093, 0.1129)	0.1113 (0.1095, 0.1131)	0.1197 (0.1178, 0.1214)	7.70 (2.53×10^{-10})
TG	0.1315 (0.1296, 0.1337)	0.1319 (0.1299, 0.1341)	0.1440 (0.1420, 0.1462)	9.58 (1.41×10^{-19})
Avg.	0.1451	0.1455	0.1940	23.72
Median	0.1135	0.1139	0.1372	16.08

Supplementary Table 18: **DeepNull improves phenotype prediction in terms of Pearson's R^2.** We evaluated three models, where each includes `age`, `sex`, and `genotyping_array` as covariates. The "Baseline" model includes a PRS computed using PLINK ($\texttt{PRS}_{\texttt{baseline}}$) and linear effect of covariates to phenotype ($\texttt{PRS}_{\texttt{baseline}}$+ Linear covariates). The "DeepNull-Baseline" model includes a PRS computed in the same way except using association results from DeepNull ($\texttt{PRS}_{\texttt{DeepNull}}$+ Linear covariates), and "DeepNull" is a model that includes both the DeepNull-based PRS and the DeepNull prediction (non-linear effect of covariates to phenotype). Here, we show the 95% confidence intervals for the squared Pearson's correlation between the model-predicted value and the true value. We see that Baseline and "DeepNull PRS" have similar performance with no statistically significant differences. However, the inclusion of the covariate non-linear prediction produces a statistically significant improvement when compared with Baseline or "DeepNull PRS". We compute the overall improvement in four ways: 1) Averaging the relative improvement, which is computed by taking the average of Δ over 10 traits, is 23.72% or 2) Computing the relative improvement over averaged R^2 of Baseline and DeepNull. In this case, we have an averaged R^2 of 0.1451 for Baseline while DeepNull has an averaged R^2 of 0.1940, this indicates a 33.65% improvement. 3) Median of the relative improvement, which is computed by taking the median of Δ over 10 traits, is 16.08% or 4) Computing the relative improvement over median of R^2 for Baseline and DeepNull. In this case, we have median R^2 of 0.1135 for Baseline while DeepNull has an median R^2 of 0.1139, this indicates a 20.88% improvement.

Pheno	#Hits	#Loci	R^2	S-LDSC Intercept	S-LDSC SNP-heritability
ALP	1695	335	0.1363 (0.1323, 0.1406)	1.0721 (0.0713)	0.1901 (0.0192)
ALT	378	174	0.1068 (0.1035, 0.1097)	1.0442 (0.0223)	0.0862 (0.0066)
AST	343	143	0.0626 (0.0598, 0.0655)	1.0335 (0.0193)	0.0612 (0.0054)
ApoB	1211	205	0.1341 (0.1323, 0.1356)	1.0726 (0.0299)	0.1300 (0.0131)
Calcium	736	272	0.0847 (0.0831, 0.0862)	1.0987 (0.0312)	0.1279 (0.0079)
GRP	34	33	0.6265 (0.6078, 0.6419)	0.9970 (0.0090)	0.0193 (0.0123)
LDL	980	202	0.1258 (0.1238, 0.1276)	1.0571 (0.0268)	0.1156 (0.0113)
Phosphate	663	229	0.1171 (0.1151, 0.1189)	1.0436 (0.0245)	0.1321 (0.0089)
SHBG	1083	319	0.2450 (0.2424, 0.2471)	1.1676 (0.0798)	0.1533 (0.0138)
TG	1251	266	0.1408 (0.1388, 0.1429)	1.1138 (0.0406)	0.1570 (0.0128)

Supplementary Table 19: **Baseline model with Spline fitting.** Here, we show the 95% confidence intervals for the squared Pearson's correlation measuring the correlation between the models predicted value and the true values. We compute the S-LDSC intercept and SNP-heritability. We observe that for all phenotypes the S-LDSC intercept is not significantly different from one which indicates no confounding. Values in parentheses for S-LDSC intercept and SNP-heritability are the standard error of the mean (s.e.m).

Pheno	SVM		Random Forest		XGBoost		DeepNull	
	MSE	MAE	MSE	MAE	MSE	MAE	MSE	MAE
ALT	189.951	8.766	180.712	8.209	180.593	8.206	180.6	8.206
ALP	669.863	17.876	659.424	17.490	659.172	17.486	658.9	17.458
Apob	0.058	0.189	0.0545	0.185	0.054	0.185	0.054	0.185
AST	109.712	5.297	106.650	5.521	106.645	5.520	106.725	5.535
Calcium	0.040	0.163	0.008	0.071	0.008	0.071	0.008	0.071
GRP	0.004	0.023	0.001	0.012	0.001	0.011	0.001	0.013
LDL	0.716	0.674	0.716	0.673	0.715	0.672	0.717	0.672
Phosphate	0.055	0.186	0.024	0.122	0.024	0.122	0.023	0.122
SHBG	646.4755	17.807	627.229	18.12	626.641	18.1068	631.372	18.167
Triglycerides	1.004	0.747	0.981	0.711	0.981	0.711	0.981	0.713

Supplementary Table 20: **Comparison of SVM (support vector machine), Random Forest, XG-Boost, and DeepNull on 10 UKB phenotypes.** We reported the MSE (mean squared error) and MAE (mean absolute error) among the methods.

Pheno	#Hits	#Loci	R^2	S-LDSC Intercept	S-LDSC SNP-heritability
ALP	1755	349	0.1572 (0.1526, 0.1625)	1.0721 (0.0713)	0.1901 (0.0192)
ALT	379	173	0.1127 (0.1095, 0.1158)	1.0442 (0.0223)	0.0862 (0.0066)
AST	351	145	0.0648 (0.0620, 0.0678)	1.0335 (0.0193)	0.0612 (0.0054)
ApoB	1230	212	0.1498 (0.1478, 0.1516)	1.0726 (0.0299)	0.1300 (0.0131)
Calcium	744	283	0.0912 (0.0893, 0.0929)	1.0987 (0.0312)	0.1279 (0.0079)
GRP	38	37	0.7312 (0.7167, 0.7433)	0.9970 (0.0090)	0.0193 (0.0123)
LDL	994	206	0.1350 (0.1332, 0.1370)	1.0571 (0.0268)	0.1156 (0.0113)
Phosphate	674	229	0.1215 (0.1195, 0.1232)	1.0436 (0.0245)	0.1321 (0.0089)
SHBG	1121	325	0.2636 (0.2608, 0.2657)	1.1676 (0.0798)	0.1533 (0.0138)
TG	1258	267	0.1437 (0.1417, 0.1458)	1.1138 (0.0406)	0.1570 (0.0128)

Supplementary Table 21: **Baseline model with XGBoost fitting.** Here, we show the 95% confidence intervals for the squared Pearson's correlation measuring the correlation between the models predicted value and the true values. We compute the S-LDSC intercept and SNP-heritability. We observe that for all phenotypes the S-LDSC intercept is not significantly different from one which indicates no confounding. Values in parentheses for S-LDSC intercept and SNP-heritability are the standard error of the mean (s.e.m).
