# Supplementary material for: DeepNull models non-linear covariate effects to improve phenotypic prediction and association power
Source: Nat Commun. 2022 Jan 11;13:241. doi: 10.1038/s41467-021-27930-0 (PMC8752755; doi:10.1038/s41467-021-27930-0)
Supplement: Supplementary file 3 — Description of Additional Supplementary Files [file 41467_2021_27930_MOESM3_ESM.pdf]

Description of Additional Supplementary Files

Title: Supplementary Data 1

Description: Comparison of Baseline and DeepNull models with only linear effects of covariates to phenotype. Pheno: Phenotyp name, DeepNull Power: DeepNull statistical power, DeepNull Expected [X^2] Causal Chrom: DeepNull expected Chi-square statistics computed from chromosome 22 where the causal variants where implanted, DeepNull Type I Error: Type I error computed for DeepNull, DeepNull Expected [X^2] Non-Causal Chrom: DeepNull expected Chi-square statistics computed from chromosomes 1 and 2, Baseline Power: Baseline statistical power, Baseline Expected [X^2] Causal Chrom: Baseline expected Chi-square statistics computed from chromosome 22 where the causal variants where implanted, Baseline Type I Error: Type I error computed for Baseline, Baseline Expected [X^2] Non-Causal Chrom: Baseline expected Chi-square statistics computed from chromosomes 1 and 2.

Title: Supplementary Data 2

Description: Comparison of Baseline and DeepNull models with covariate interactions. Files have the same column names as Supplementary Data 1.

Title: Supplementary Data 3

Description: Adjusting for covariates provided to DeepNull during association testing is necessary to avoid bias. x: Random variable representing genotype, yhat: Predicted value of the phenotype based on the regression model, z1: Random variable representing the confounder, z2: Random variable representing the square of the confounder, bias_x: Difference between the estimated and true coefficients for x. This represents bias in estimating the genotypic effect, bias_yhat: Difference between yhat and the true phenotype, bias_z1: Difference between the estimated and true coefficients for z1, bias_z2: Difference between the estimated and true coefficients for z2, model: Association model, described by the variables being adjusted for, beta_x: Estimated coefficient for x, beta_z1: Estimated coefficient for z1, beta_z2: Estimated coefficient for z2, config: Arbitrary index for the simulation configuration, reps: Number of simulation replicates, rho: Correlation between x and z1. In the absence of correlation, i.e. rho = 0, z1 is not a confounder, sample_size: Number of independent subjects simulated on each simulation replicate.
