# Supplementary material for: DeepNull models non-linear covariate effects to improve phenotypic prediction and association power
Source: Nat Commun. 2022 Jan 11;13:241. doi: 10.1038/s41467-021-27930-0 (PMC8752755; doi:10.1038/s41467-021-27930-0)
Supplement: Supplementary file 7 — Reporting Summary [file 41467_2021_27930_MOESM7_ESM.pdf]

nature research

Corresponding author(s): Cory Y. McLean and Farhad Hormozdiari

Last updated by author(s): Nov 12, 2021

Reporting Summary

Nature Research wishes to improve the reproducibility of the work that we publish. This form provides structure for consistency and transparency in reporting. For further information on Nature Research policies, see our Editorial Policies and the Editorial Policy Checklist.

Statistics

For all statistical analyses, confirm that the following items are present in the figure legend, table legend, main text, or Methods section.

n/a	Confirmed	
☐	☒	The exact sample size (*n*) for each experimental group/condition, given as a discrete number and unit of measurement
☒	☐	A statement on whether measurements were taken from distinct samples or whether the same sample was measured repeatedly
☐	☒	The statistical test(s) used AND whether they are one- or two-sided<br>*Only common tests should be described solely by name; describe more complex techniques in the Methods section.*
☐	☒	A description of all covariates tested
☐	☒	A description of any assumptions or corrections, such as tests of normality and adjustment for multiple comparisons
☐	☒	A full description of the statistical parameters including central tendency (e.g. means) or other basic estimates (e.g. regression coefficient) AND variation (e.g. standard deviation) or associated estimates of uncertainty (e.g. confidence intervals)
☐	☒	For null hypothesis testing, the test statistic (e.g. *F*, *t*, *r*) with confidence intervals, effect sizes, degrees of freedom and *P* value noted<br>*Give P values as exact values whenever suitable.*
☒	☐	For Bayesian analysis, information on the choice of priors and Markov chain Monte Carlo settings
☒	☐	For hierarchical and complex designs, identification of the appropriate level for tests and full reporting of outcomes
☐	☒	Estimates of effect sizes (e.g. Cohen's *d*, Pearson's *r*), indicating how they were calculated

Our web collection on statistics for biologists contains articles on many of the points above.

Software and code

Policy information about availability of computer code

Data collection	No software is used for data collection. We have used available UK Biobank data.
Data analysis	BOLT-LMM software (v2.3.5): https://data.broadinstitute.org/alkesgroup/bolt-lmm<br>BaselineLD annotations: https://data.broadinstitute.org/alkesgroup/ldscore<br>DeepNull software: github.com/google-health/genomics-research/nonlinear-covariate-gwas [https://doi.org/10.5281/zenodo.5636318]<br>GWAS Catalog: https://www.ebi.ac.uk/gwas/<br>PLINK software (v1.9): https://www.cog-genomics.org/plink1.9<br>TensorFlow (v 2.4.1): https://www.tensorflow.org<br>UK Biobank study: https://www.ukbiobank.ac.uk<br>XGBoost: https://xgboost.readthedocs.io/en/latest/<br>UCSC LiftOver: https://genome.ucsc.edu/cgi-bin/hgLiftOver

For manuscripts utilizing custom algorithms or software that are central to the research but not yet described in published literature, software must be made available to editors and reviewers. We strongly encourage code deposition in a community repository (e.g. GitHub). See the Nature Research guidelines for submitting code & software for further information.

Data

Policy information about availability of data

All manuscripts must include a data availability statement. This statement should provide the following information, where applicable:

- Accession codes, unique identifiers, or web links for publicly available datasets
- A list of figures that have associated raw data
- A description of any restrictions on data availability

UK Biobank study:https://www.ukbiobank.ac.uk and our access was approved under Application 65275.

Field-specific reporting

Please select the one below that is the best fit for your research. If you are not sure, read the appropriate sections before making your selection.

☒ Life sciences ☐ Behavioural & social sciences ☐ Ecological, evolutionary & environmental sciences

For a reference copy of the document with all sections, see nature.com/documents/nr-reporting-summary-flat.pdf

Life sciences study design

All studies must disclose on these points even when the disclosure is negative.

Sample size	We use all the samples provided by UK Biobank. We utilize all the European samples in our analysis.
Data exclusions	We removed samples with excess heterozygosity, missingness, or putative sex chromosome aneuploidy as defined by Bycroft et al 2018 (https://www.nature.com/articles/s41586-018-0579-z). We further limited to individuals of European genetic ancestry as defined in Alipanahi et al 2021 (https://doi.org/10.1016/j.ajhg.2021.05.004)
Replication	We have used GWAS catalog to replicate our GWAS finding.
Randomization	We use all the samples provided by UK Biobank. We only randomized the data in the process of 5-fold cross validation. However, this is done in our DeepNull code and we have used the exact seed as provided in the Github so the result is replicated.
Blinding	No blinding has been performed in our analysis. We use all the samples provided by UK Biobank.

Reporting for specific materials, systems and methods

We require information from authors about some types of materials, experimental systems and methods used in many studies. Here, indicate whether each material, system or method listed is relevant to your study. If you are not sure if a list item applies to your research, read the appropriate section before selecting a response.

Materials & experimental systems

n/a	Involved in the study
☒	☐ Antibodies
☒	☐ Eukaryotic cell lines
☒	☐ Palaeontology and archaeology
☒	☐ Animals and other organisms
☒	☐ Human research participants
☒	☐ Clinical data
☒	☐ Dual use research of concern

Methods

n/a	Involved in the study
☒	☐ ChIP-seq
☒	☐ Flow cytometry
☒	☐ MRI-based neuroimaging
